# How choice and motor mimicry affect affiliation: An fNIRS study

**DOI:** 10.1162/IMAG.a.40

**Published:** 2025-06-16

**Authors:** Paula Wicher, Eva G. Krumhuber, Aiko Murata, Sabina Beganovic, Antonia F. de C. Hamilton

**Affiliations:** UCL Institute of Cognitive Neuroscience, University College London, London, United Kingdom; Department of Experimental Psychology, University College London, London, United Kingdom; NTT Communication Science Laboratories, Nippon Telegraph and Telephone Corporation, Atsugi, Japan

**Keywords:** fNIRS, mimicry, social interaction, affiliation, homophily, mirror neuron system

## Abstract

It is widely believed that being mimicked causes liking and social bonding, but the underlying neural mechanisms remain largely unknown. Additionally, it is unclear whether similar effects arise when abstract choices or physical actions are mimicked. In this study, we used functional near-infrared spectroscopy (fNIRS) to examine the involvement of temporal and parietal brain regions during in-person interactions in which participants were either mimicked by a confederate in terms of their motor movements (*n*= 30) or their choices (*n*= 30). In the Choice group, the participant and confederate chose the same (mimicry) or different (no mimicry) paintings in the absence of any physical mirroring action by the confederate. In the Motor group, the participant and confederate performed the same (mimicry) or different (no mimicry) right-hand movements while viewing distinct pairs of pictures. Choice mimicry led to a strong sense of affiliation across various measures, which was evident through activation of the bilateral inferior parietal lobule (IPL). In contrast, motor mimicry produced only a subtle liking effect and showed activation in the left posterior superior temporal sulcus (STS) and the left temporo-parietal junction (TPJ). In addition, awareness of the study’s goal increased the right intraparietal sulcus (IPS) activity in the Choice group but did not do so in the Motor group. These findings suggest that choice mimicry exerts a stronger influence on affiliation than motor mimicry and highlight differential neural pathways for detecting mimicked choices and mimicked actions.

## 1 Introduction

If Anna copies the actions of Beth, it is widely believed that Beth will then like Anna more. That is, Anna’s mimicry acts as a “social glue” that induces Beth to like her (Chartrand & Bargh, 1999;Chartrand & Lakin, 2013). This phenomenon is intriguing and potentially very important to human social bonding, but it is also hard to pin down in controlled laboratory experiments. Natural mimicry arises spontaneously during a social interaction, which means opportunities to capture and measure the consequences of mimicry are limited, in particular in relation to brain imaging measures. The present paper represents a first attempt to develop a new task that creates opportunities for participants to be mimicked in a naturalistic setting but with enough experimental control to allow brain imaging data to be collected. We aim to track the neural mechanisms that underlie the effect of being mimicked and to understand how this links to social bonding.

Spontaneous social mimicry of motor movements, such as gestures or postures, has often been described as a social glue, fostering connections between individuals. This behaviour has been shown to induce positive effects on the person being mimicked, including increased liking (Chartrand & Bargh, 1999;Chartrand & Lakin, 2013), more favourable perceptions of the mimicker (Bretter et al., 2024;Gueguen et al., 2009), and enhanced prosocial tendencies (Stel et al., 2011). Nevertheless, attempts to replicate these positive outcomes have often been inconsistent, with effect sizes that tend to be small (Hale & Hamilton, 2016a,2016b;Majka et al., 2020;Rauchbauer et al., 2020;van Swol, 2003). Furthermore, recent research has broadened the focus to include less traditional forms of mimicry, such as lexical (Lelonkiewicz et al., 2021), syntactic mimicry (Abrahams et al., 2018), and human–computer interaction mimicry (Arakawa & Yakura, 2020). To date, our understanding of how different types of mimicry vary in their ability to induce liking remains limited.

In recent work we explored mimicry in more abstract domains by investigating the effects of being mimicked in terms of preferences (Wicher et al., 2024). To clearly differentiate the effect of being mimicked by another person from the effect of producing mimicry, we introduce the term “BeMim” to specifically refer to the former. Choice BeMim is defined as the experience of another person copying one’s choices, such as preferences for art paintings (Wicher et al., 2025). In contrast, Motor BeMim refers to the experience of another person copying one’s motor actions, such as hand movements. Our behavioural studies show that Choice BeMim led to a robust liking effect, whereas Motor BeMim may not do so (Wicher et al., 2024). These results extend prior work on children, which showed that Choice BeMim enhanced trust (Over et al., 2013) and affiliation (Gerson et al., 2017) towards the mimicker. Building on this work, the current study aims to compare the impact of being mimicked in motor movements versus choices during face-to-face interactions, with a between-groups experimental design and the addition of fNIRS brain imaging to track neural mechanisms of BeMim effects.

Despite the growing body of behavioural research, much remains unknown about the cognitive and neural mechanisms underlying the state of being mimicked, as opposed to mimicking others (Hale & Hamilton, 2016b;Wicher et al., 2025). Therefore, a second goal of the study was to investigate the neural mechanisms involved in Choice BeMim and Motor BeMim using functional near-infrared spectroscopy (fNIRS). Current research offers only tentative suggestions about the brain regions involved in people’s responses to being mimicked. The following sections review these studies to allow us to set up hypotheses, with a focus on regions accessible to fNIRS as applied in this study.

### 1.1 Candidate neural mechanisms of Motor BeMim

A strong candidate for neural systems involved in Motor BeMim is the mirror neuron system (MNS), which includes the posterior part of the inferior frontal gyrus (IFG) and the premotor cortex, as well as the inferior parietal lobe (IPL) and intraparietal sulcus (IPS) (Contaldo et al., 2016;Hale & Hamilton, 2016b;Rizzolatti & Craighero, 2004). Only the latter parietal regions are accessible to our fNIRS in this study. All these regions are well established in terms of their roles in mimicry production, particularly for producing, observing, and imitating actions (Caspers et al., 2010;Grèzes & Decety, 2001;Iacoboni et al., 1999).Decety et al. (2002)performed a PET study including overt BeMim (and mimicry production). The experimenter and participant each made right-hand movements directed at three small objects and could see each other’s hands on a video monitor. Results showed that the left IPL was more active during mimicry production, while the right IPL was more engaged in recognising others’ motor actions as similar when being mimicked. In addition,Miyata et al. (2021)used fMRI to study participants as they engaged in explicit turn-taking to imitate each other’s facial movements via real-time video streaming in the scanner. Their results revealed activation in the right IPL and right IFG during the explicit experience of being mimicked, further supporting the involvement of the MNS.

A second process closely linked to the detection of BeMim is self-other differentiation, that is, knowing whether observed movements are self- or other-related (Brass et al., 2009). This can be linked to activity in the temporoparietal junction (TPJ) and the superior temporal sulcus (STS). In an fMRI study byBrass et al. (2009), participants performed index or middle finger movements that were congruent or incongruent with an image of a hand making finger movements either before (participant copies) or after (participant experiences BeMim) the participant’s own finger movement. TPJ activation occurred when participants were being mimicked and when they observed incongruent stimuli, suggesting that the TPJ acts as a broad mechanism for detecting mismatches, such as visuo-motor discrepancies between self and others (Sperduti et al., 2011).Miyata et al. (2021)found activation of both TPJ and STS in explicit BeMim of facial actions during fMRI and linked this to self-other differentiation. This means that TPJ and STS are strong candidates for distinguishing between actions of self and other in the context of Motor BeMim.

### 1.2 Candidate neural mechanisms of Choice BeMim

Choice BeMim effects arise when a participant makes a choice (e.g., indicates which painting they prefer) and then a confederate chooses the same painting, and a small number of neuroimaging studies have explored similar tasks. A study fromFarmer et al. (2019)used a related design in fMRI. On each trial, the participant chose a painting and then saw the choices of a similar agent who chose the same on 75% of trials and a dissimilar agent who chose the same on only 25% of trials; note the participants believed that the agents made independent choices and had not seen (could not mimic) the participant’s choice. There was increased activation in the right angular gyrus (AG), the right IPS, and the right STS when the agent’s choice was inconsistent with participants’ own behaviour. A different approach with relevance to Choice BeMim was used byCampbell-Meiklejohn et al. (2010). After rating a set of songs, participants in fMRI saw the preferences of two “experts” who either agreed or disagreed with the ratings; then participants re-rated the songs. Results showed that right TPJ was involved in monitoring others’ choices, with greater activation in participants who were more influenced by the experts’ preferences. In a study bySuzuki et al. (2015)on consensus decision making, there was increased activity in the right STS/TPJ when the majority of the group choices aligned with the participant’s own selection, while bilateral activation in the IPL and IPS tracked the extent to which each choice was maintained by other group members.

Although the above studies on choice-related social influence lacked real social interactions and did not explore cases where the participant’s choice is explicitly mimicked by another, the results can still guide our predictions about Choice BeMim. All the findings suggest that TPJ, STS, and IPS are strong candidate regions for relating self-choices to the choices made by others, and thus we can predict that the same areas may be involved in our BeMim task.

### 1.3 The current study

The current study aimed to explore the neural and cognitive mechanisms underlying Choice and Motor BeMim. For this, we used fNIRS to track participants’ temporal and parietal brain activity while they were being mimicked. Participants were placed in the context of an art choice task where, on each trial, they must indicate which of two paintings they prefer and see a confederate indicate a preference (Farmer et al., 2019;Forbes et al., 2019). Two groups of participants experienced two different types of mimicry. In the Choice mimicry group, participant and confederate saw matching paintings, and the participant indicated a choice with a pointing movement while the confederate’s choice was indicated verbally; this meant only the abstract choice and not the action was shared. In the Motor mimicry group, participant and confederate had different paintings; the participant indicated a choice with a pointing movement and the confederate also used a pointing movement to indicate their choice from the other pair of paintings; this meant that motor actions were shared but abstract choices were not.

Each participant performed two of these mimicry induction blocks (30 trials each) with two different confederates, one who mimicked on the majority of trials and one who did not. We refer to these blocks as the BeMim condition and the No-BeMim condition. After each block, participants rated the confederate on affiliation, including perceived warmth and competence. fNIRS data were recorded throughout the induction blocks and ratings. At the end of the study, participants completed additional measures of their preference for one of the two confederates, and completed a forced-choice behavioural intention task and the Maze Game (Hale et al., 2018), as well as various trait questionnaires. The mixed design allowed for the examination of behavioural, neural, and cognitive processes involved in Choice and Motor BeMim, enabling comparison between the two forms of mimicry and providing an assessment at the individual level.

Based on our prior work and the literature outlined above, we can draw out hypotheses for both our behavioural ratings and our brain imaging data. First, we predict that, in both the Motor and Choice groups, participants should report higher liking for the BeMim confederate compared with the No-BeMim confederate. This will be indicated in higher ratings of affiliation and warmth as well as more choices of the BeMim confederate in the behavioural intentions tasks (that force participants to choose between the two confederates) and in the Maze Game. We do not have specific predictions for competence-related items. Second, we predict that these BeMim effects should be stronger in the Choice group than in the Motor group, in line withWicher et al. (2024). Given its exploratory nature, this study was not preregistered.

In terms of brain imaging data, we predict that the Motor BeMim effects may be linked to engagement of parietal MNS regions (IPL, AG, SMG, IPS). We further predict that Choice BeMim effects may be more linked to the perspective-taking network (TPJ, STS). This distinction between motor effects in the MNS and abstract-preference effects in TPJ and STS aligns with the literature outlined above where motor mimicry tasks engage different brain regions to abstract perspective tasks.

## 2 Methods

### 2.1 Participants and confederates

We conducted an a-priori sample size calculation using a mixed-effects model to determine the necessary sample size for our mixed-design study considering perceived warmth as the outcome, two groups (Choice vs. Motor) and two conditions (BeMim vs. No-BeMim). Based on previous research (Wicher et al., 2024), we estimated an effect size of Cohen’s*d*= .65. Using the*pwr*package in R for data analysis, we calculated that a minimum of 30 participants per group would be necessary for a desired power level of 0.90, resulting in a total of 60 participants.

To ensure a sufficient number of valid data points, we collected data from 65 participants from a local participant database. Eligibility criteria included being over 18 years old, fluency in English, absence of fixed hairstyles (e.g., extensions) that could interfere with the fNIRS head cap, and normal or corrected-to-normal vision. Two participants were excluded, one due to technical issues and another due to limited English proficiency. The behavioural sample included in the analysis comprised 63 participants (54 females, 9 males) with a mean age of 23.4 years (*SD*= 4.04, range 19–38). The participants were from diverse backgrounds: 48% China, 19% European Union, 17% United Kingdom, and 16% other countries. The final fNIRS sample consisted of 60 participants (seeSection 2.5.2for further details).

Five female confederates, all UCL master’s students majoring in non-psychology disciplines (*M*_age_= 24.4,*SD*= 2.9), were hired for the study. Among them, four were of Chinese origin and one was Italian. They were assigned stage names (Anna, Beth, Claire, Diana, and Ellie) for their interactions with participants. The confederates were kept unaware of the study’s aims and hypotheses. They were instructed to act as though they were participants themselves and to avoid disclosing their familiarity with the procedure. They were encouraged to interact naturally with the participants using non-verbal cues by making eye contact or smiling but were instructed not to speak to the participants. All participants and confederates provided written informed consent. Participants were paid £15, while confederates received £10 per hour. The study, which lasted approximately 90 minutes, received ethical approval from the UCL Research Ethics Committee (Approval ID Number: 5975/003). Materials, analyses codes, and pre-processed data are publicly accessible via the Open Science Framework (OSF) athttps://osf.io/f6xkp/?view_only=2e6c520f656d4f86916d9d82565d08f4.

### 2.2 The art choice task

All participants completed the Art Choice Task and were randomly assigned to either the Choice or Motor groups. In both groups, the experimental procedure was identical and involved 1 BeMim block and 1 No-BeMim block with 2 different confederates with 30 trials in each block. In each BeMim block, participants and confederates completed 17 congruent trials (confederate mimics participant), 8 incongruent trials (confederate does not mimic participant), and 5 null trials out of a total of 30 trials. Conversely, in the No-BeMim block, they completed 17 congruent trials (confederate does not mimic), 8 incongruent trials (confederate mimics participant), and 5 null trials (Fig. 2D). Trial order was pseudorandomised in all blocks.

**Fig. 1.**
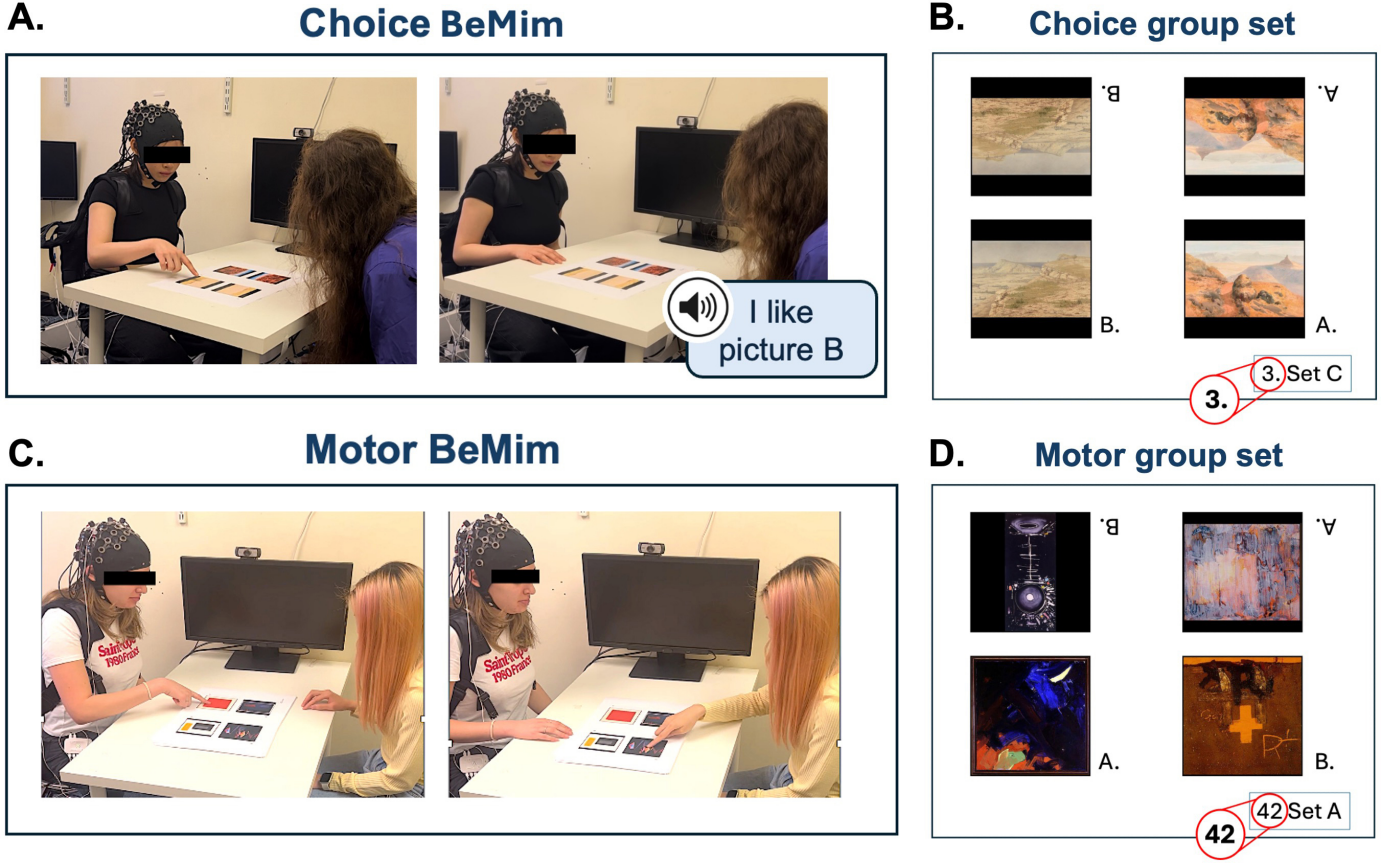
An overview of the experimental set up and picture sets for Choice and Motor groups. (A) In the Choice group, the participants pointed to their preferred picture, while the confederates pressed a button on a keyboard on their lap to generate a voice command indicating their choice. In the Choice BeMim trial, both the participant and the confederate chose the same picture, while in the Choice No-BeMim trial, the confederate selected the opposite image to the participant. (B) An example of a picture set for the Choice group, consisting of two identical pairs of pictures, with each pair facing the person making the choice. Confederates were instructed to select the same picture as the participant if a full stop was present next to the picture sheet number, and to choose the opposite picture if no full stop was present. (C) In the Motor group, both the participant and the confederate pointed to the selected image using their right hand. In the Motor BeMim trials, they performed the same right-hand action, while in the Motor No-BeMim trials, they performed different right-hand actions. (D) An example of a picture set for the Motor group, consisting of two distinct pairs of images, with each pair facing the person making the choice. Confederates were instructed to point to the picture with the same letter as the participant using their right hand if a full stop was present next to the page number, and to select the opposite letter with their right hand if no full stop was present.

For the Choice group, a picture set was prepared with each page showing a pair of pictures oriented to the participant and the same pair oriented to the confederate (Fig. 1B). On each trial, the participants pointed to their preferred picture, then the confederates pressed a button on a keyboard on their lap to generate a voice command indicating their choice (Fig. 1A). In both BeMim-Congruent trials and No-BeMim-Incongruent trials, the confederate selected the same picture as the participant, thus mimicking their choice. In both BeMim-Incongruent trials and No-BeMim-Congruent trials, the confederate selected the opposite picture from the participant and did not mimic their choice. This setup, using different motor modalities for the participant and the confederate (pointing vs. pressing a button), allowed the induction of Choice BeMim without any motor mimicry. To reduce cognitive load on confederates, they were cued on each trial whether to mimic or not by the presence or absence of a full stop in the number on each picture sheet (Fig. 1B). During the null trials, a single pair of images was presented only to the participant (not the confederate), enabling the participant to select a picture without any confederate’s response, and these trials lasted as long as the other trials.

For the Motor group, the picture set consisted of two distinct pairs of images with labels (A and B), such that if the participant’s picture A was on her left, the confederate’s picture A was also on their own left (Fig. 1D). On each trial, the participants pointed to a preferred picture using their right hand and then the confederate pointed to a picture (Fig. 1C). In both BeMim-Congruent trials and No-BeMim-Incongruent trials, the confederate used exactly the same motor action as the participant; if the participant made an ipsilateral movement to the picture nearest their right shoulder, the confederate also made an ipsilateral movement to the picture nearest her own right shoulder; if the participant made a contralateral movement to the picture nearest her left shoulder, the confederate also made a contralateral movement to the picture nearest her own left shoulder. Thus, in motor mimicry conditions, the confederate shows anatomical imitation of the participant using the same muscles and arm kinematics as the participant. In both BeMim-Incongruent and No-BeMim-Congruent trials, the confederate used the other possible action not used by the participant; if the participant makes an ipsilateral movement, then the confederate makes a contralateral movement and vice versa. Again, to reduce cognitive load on confederates, they were cued on each trial whether to mimic or not by the presence or absence of a full stop in the number on each picture sheet (Fig. 1D).Supplementary Figure S1, which illustrates all BeMim and No-BeMim trial variations for both the Choice and Motor groups, is included in theSupplementary Materials.

During the experiment, participants were instructed to always place their right hand on the table and their left hand on their lap. They were asked to indicate their preference in the Art Choice Task by pointing, using only the index finger of their right hand, and tapping the chosen picture without speaking. This approach ensured that the fNIRS signal exclusively captured BeMim responses, avoiding potential interference from language factors. Throughout these interactions, brain activity in the temporal and parietal cortex was continuously recorded using a Shimadzu LightNIRS device (seeSection 2.5for further details).

#### 2.2.1 Timeline of one trial

During each 20-second trial, the participant always made the decision first, followed by the confederate’s choice. Trials were precisely timed using voice commands controlled by MATLAB Psychtoolbox, ensuring consistent timing for accurate fNIRS data recording. After the command “Trial start”, there was a 3-second pause. The participant then heard “Which picture do you like?” and had 5 seconds to familiarise themselves with the pair of pictures in front of them. Next, the command “Participant one” indicated it was the participant’s turn to point with their right hand to their preferred picture. The command “Participant two” signalled to the confederate that it was their turn to make a choice. Both the participant and confederate had 4 seconds each to respond after their respective prompts. The trial concluded with the “Trial end” command. A brief 1- to 2-second pause between trials allowed the research assistant to remove the top sheet of pictures before starting the next trial. For the fNIRS analysis, the onset for BeMim and No-BeMim trials was defined from the “Participant two” command to “Trial end”. The baseline was calculated across all trials (BeMim, No-BeMim, and null) from “Which picture do you like?” to “Participant two” command (seeSection 2.6for further details). Detailed timing for these trials can be found inFigure 2Bfor the Choice group andFigure 2Cfor the Motor group.

**Fig. 2.**
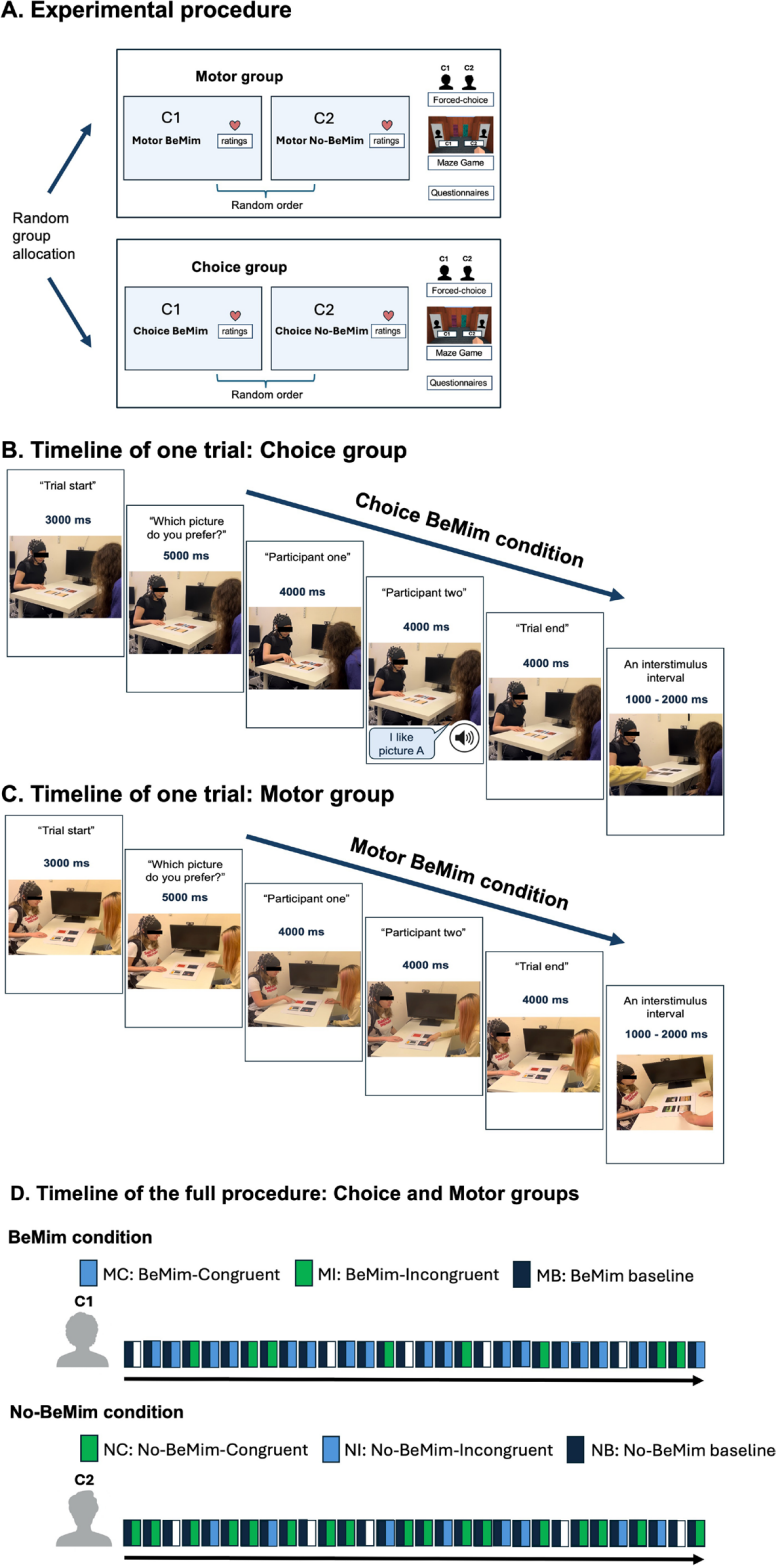
An overview of the experimental procedure and trial timelines for Choice and Motor groups. (A) Before the experiment, participants were randomly allocated to either the Motor or Choice group. In both groups, each participant engaged in the Art Choice task with two different confederates. After each condition, participant provided person perception ratings. At the end, participant answered forced-choice questions about their behavioural intentions, and completed the Maze Game as well as several questionnaires. (B) Trial timeline for the Choice group, precisely timed using voice commands. (C) Trial timeline for the Motor group, also precisely timed by voice commands. (D) Full procedure timeline for both the Choice and Motor groups. In the BeMim condition, participants and confederates experienced 17 BeMim-Congruent trials, 8 BeMim-Incongruent trials, and 5 BeMim null trials, for a total of 30 trials. Conversely, in the No-BeMim condition, they experienced 17 No-BeMim-Congruent trials, 8 No-BeMim-Incongruent trials, and 5 No-BeMim null trials. For BeMim and No-BeMim trials (MC, MI, NC, NI), brain responses were analysed from the “Participant two” command to the end of each trial. Baseline brain responses were calculated across all trial types from the “Which picture do you like?” command until “Participant two”.

### 2.3 Procedure

Data were collected in person at the Institute of Cognitive Neuroscience in London, UK. Before visiting the laboratory, participants were randomly assigned to the Choice or Motor group. After signing a consent form, participants were told that they would participate in an art game designed to explore new interactive ways of experiencing art with two other participants. For this, they would need to make a selection by pointing to one of the two paintings they preferred. The other participants would then indicate their choice. They completed a short training phase of two trials with a research assistant. As part of the cover story, participants were told that, due to a lack of devices, they had been selected from among three participants to wear the fNIRS cap for measuring brain response to art. The researcher and the research assistant then prepared the participant for the experiment by fitting them with the fNIRS cap. The Shimadzu LightNIRS device was placed in a backpack and positioned on the participant’s back (seeSection 2.5for further details). In addition, a Plux BioSignals system was used to record ECG (heart-rate) with electrodes attached to the left side of the participant’s chest. To ensure comprehensive coverage, three laboratory cameras recorded the experiment: one overhead capturing both the confederate and participant making choices, the second focused on the participant, and the third on the confederate.

Following this preparation, confederate A entered the room and took a seat at the table in front of the participant (Fig. 1A, C). They were seated 1 meter apart from each other. A set of 60 individual picture sheets was arranged on the desk between the participant and the confederate, covered with a blank sheet and positioned for easy access. The researcher then initiated the fNIRS data acquisition. The experimental procedure began with removing the blank sheet from the picture set, followed by the voice command “Trial start”. After the participant completed 30 trials with confederate A, which lasted approximately 10 to 13 minutes, confederate A left the room with the research assistant. The participant remained seated and answered a set of questions (Table 1) using a laptop in Gorilla (gorilla.sc).

**Table 1.** Summary of behavioural measures.

	Items	Question example	Scale
**Measures completed after each block**			
Current affective state	1	“How did you feel during the art game with [confederate]?”	0 ( *negative* ) - 100 ( *positive* )
Perceived warmth	5	“Do you think [confederate] is a warm person?”	1 ( *definitely not* ) - 6 ( *definitely yes* )
Perceived competence	5	“Do you think [confederate] is competent?”	1 ( *definitely not* ) - 6 ( *definitely yes* )
Perceived rapport	3	“I think [confederate] and I established rapport” ( Gratch et al., 2007 )	0 ( *negative* ) - 100 ( *positive* )
Perceived closeness	1	Measured using the Inclusion of Other in the Self (IOS) scale ( Aron et al., 1992 ).	1 ( *no overlap)* - 7 ( *most overlap)*
Perceived positive attributes	7	“To what extent does [confederate] possess this trait: [e.g., generosity]?” ( Harker & Keltner, 2001 )	1 ( *not at all* ) - 100 ( *very much* )
**Measures completed after both blocks**			
Warmth-related behavioural intentions	4	“Who would you like to go to the art gallery with?”	Forced choice (Confederate 1 / Confederate 2)
Competence-related behavioural intentions	4	“Who would you ask to help you with an essay?”	Forced choice (Confederate 1 / Confederate 2)
Maze Game - trust	12	“Who will help you in making the decision [which door to open in the maze]?” ( Hale et al., 2018 )	Forced choice (Confederate 1 / Confederate 2); Choice to accept/reject hint

Next, confederate B entered the room, and the experimental procedure was repeated, with confederate B adopting the opposite behaviour (No-BeMim or BeMim) to that of confederate A in the first condition. After completing the Choice Art Task, confederate B was escorted out of the room by the research assistant. Before the fNIRS cap was removed, participants answered the same questions as after the first block. Following both interactions, the fNIRS cap was removed and participants completed the final set of measures including forced-choice questions about behavioural intentions (Table 1). At the end of the study, participants were asked a set of debriefing questions about the study’s goal and whether they noticed anything specific about other participants. This information helped identify participants who may have guessed the study’s objective or recognised that they were interacting with confederates, as these factors could influence the study results (Kulesza et al., 2016;Manusov, 1992). Finally, participants were informed about the true purpose of the study, compensated for their participation, and thanked for their involvement. The trial order, condition order, and physical appearance of confederates were counterbalanced. In total, the experiment lasted approximately 90 minutes. A full description of all rating and behavioural intention measures (Section 2), the Maze Game used to assess social approach and trust (Section 6) are provided in theSupplementary Materials.

### 2.4 Behavioural data analysis

R software (version 4.4.0) was used for behavioural data analysis. Rating scores in the BeMim and No-BeMim conditions were analysed across the Choice and Motor groups. Perceived warmth and competence ratings were analysed separately to test for between-group (Choice vs. Motor) and within-group (condition: BeMim vs. No-BeMim) effects using linear mixed-effects models with the*lmer*function from the lme4 package in R (Bates et al., 2014). For all mixed-effects models, we used dummy coding, specifying “Choice” as the reference level for the group factor and “No-BeMim” as the reference level for the condition factor. Separate models were then applied to analyse the Choice and Motor groups independently, focusing on the within-group effect (condition). Similarly, for ratings of perceived affective state, rapport, closeness, and positive attributes, linear mixed-effects models were used, considering both between-group (Choice vs. Motor) and within-group (condition) effects. For the behavioural intentions tasks, four chi-squared tests were conducted to test whether participants preferred one confederate over the other, for the Choice and Motor groups with warmth-related and competence-related decisions separately. In addition, based on information from the debriefing, participants who both correctly identified the study’s goal and realised they were interacting with confederates were coded as 1 (and 0 otherwise). In the Choice group, 13 out of 31 participants met these criteria, while in the Motor group, 7 out of 32 did so. An exploratory logistic regression analysis was conducted to examine whether participants’ awareness differed by group (Choice vs. Motor), with the Choice group serving as the reference. Further analysis of the potential effect of awareness on warmth‐ratings scores is given inSupplementary Materials(Section 4.2).

### 2.5 fNIRS signal acquisition

**Fig. 3.**
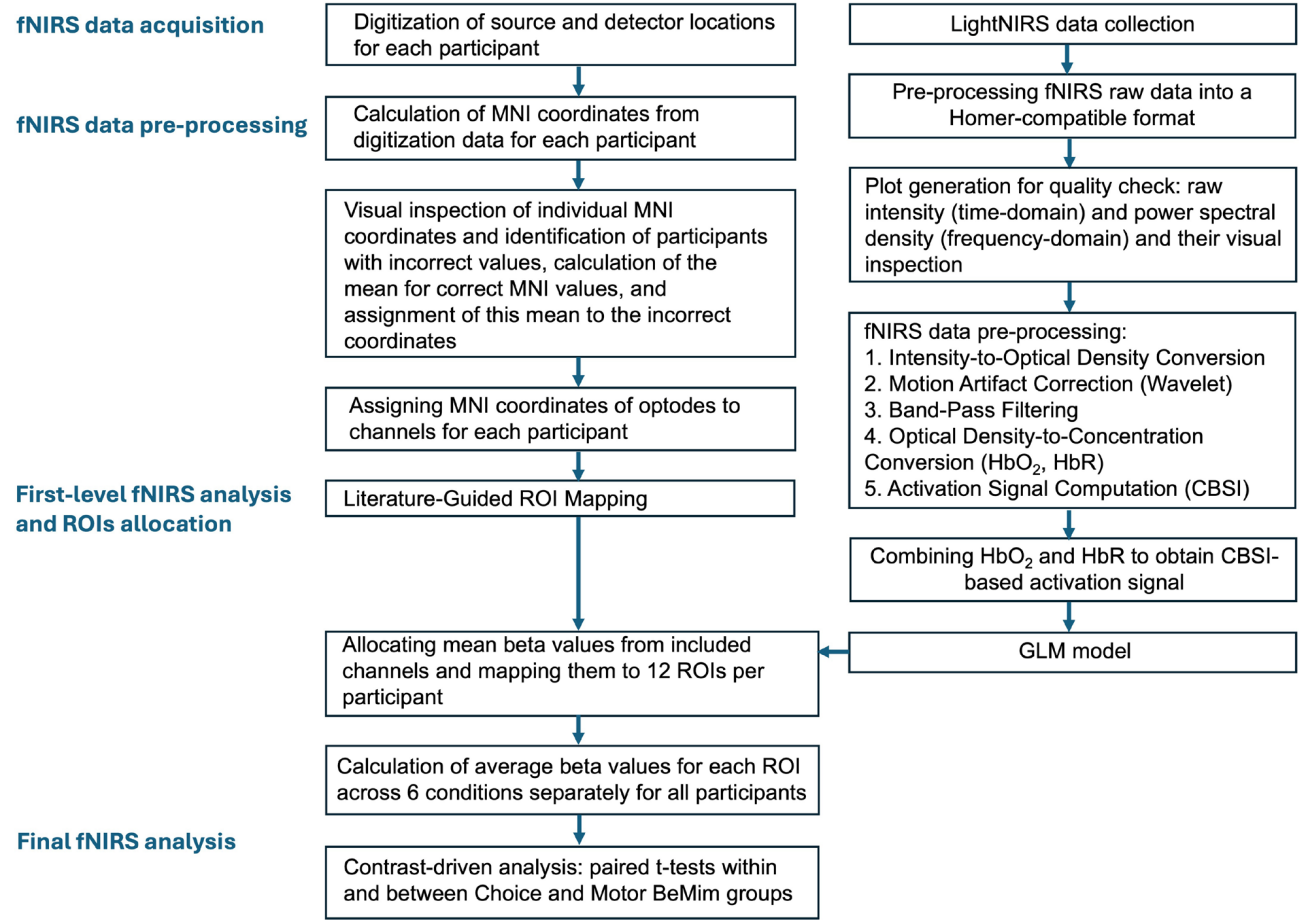
Workflow diagram of the study’s fNIRS data processing pipeline: from signal acquisition to final analysis.

**Fig. 4.**
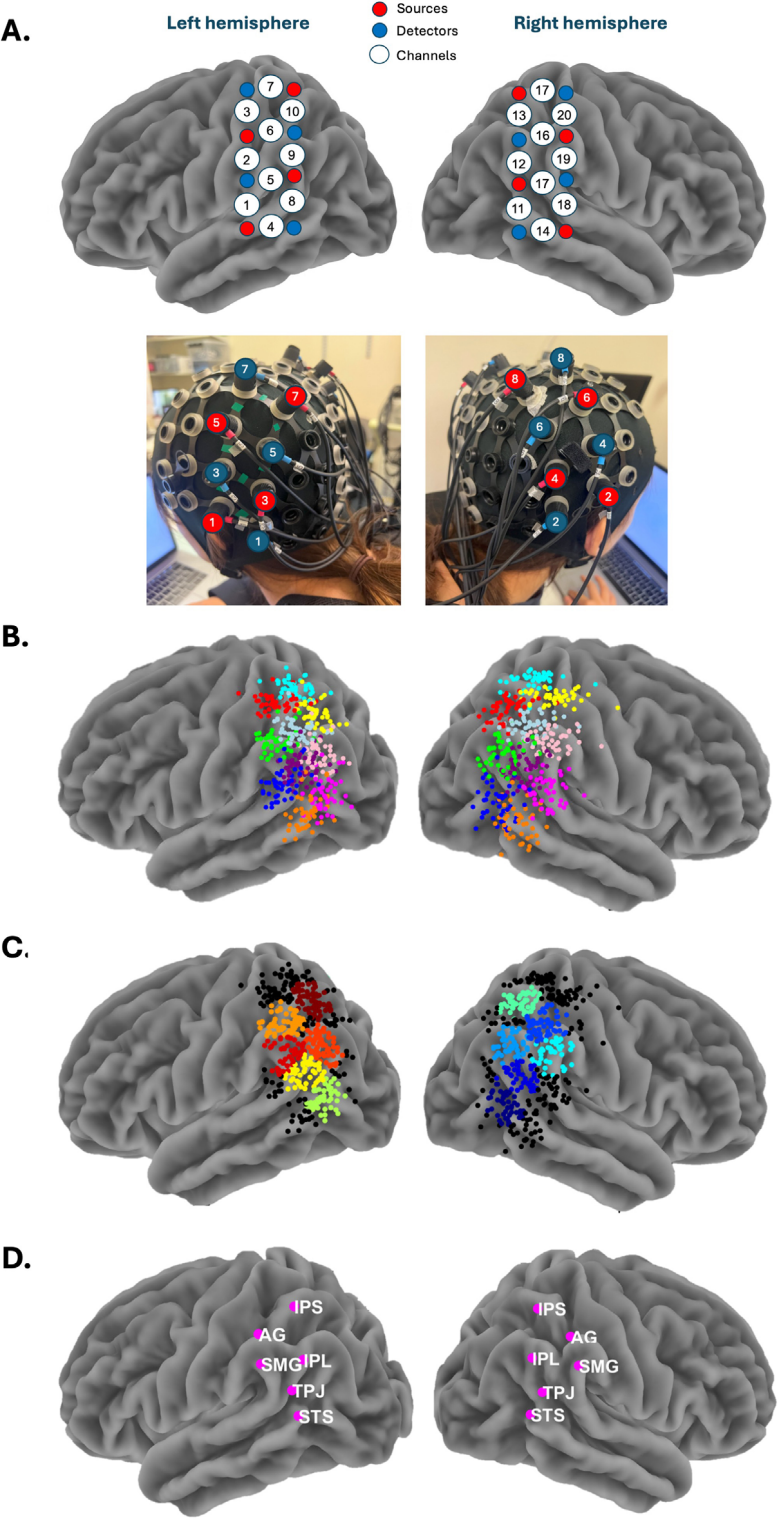
Optode configuration, fNIRS data from all participants, and selected ROIs. (A) Diagram illustrating the positions of light sources (red), detectors (blue), and the 20 channels (white) formed by the 16 probes used in the experiment. The picture below shows the locations of each light source and detector for every participant. (B) Diagram presenting data from all participants for 10 channels on the left and 10 channels on the right hemisphere, with each colour representing corresponding channel positions across the two hemispheres. (C) Diagram showing data from all participants for selected Regions of Interest (ROIs) on the left and right hemispheres, with each colour representing one ROI. Black dots indicate participants’ data that were not included in the final ROIs. (D) Diagram showing the mean values of the final ROIs used for the fNIRS data analysis in this study.

During the experiment, brain activity in the temporoparietal cortices bilaterally was recorded using a portable LIGHTNIRS fNIRS device (Shimadzu Corporation, Kyoto, Japan) with KNIRS software. Data acquisition involved 16 optodes, consisting of 8 light sources and 8 detectors, distributed across both hemispheres and using 3 wavelengths of light (780, 805, and 830 nm) at a sampling frequency of 3 Hz. Optodes were arranged to cover the parietal and posterior temporal cortex bilaterally, with a source–detector separation set to 3 cm, resulting in 20 channels of interest (Fig. 4A).Figure 3illustrates the complete pipeline for obtaining fNIRS data through preprocessing and ROI allocation to the final analysis.

#### 2.5.1 Optode localisation

For each participant, the location of vertex on the scalp was measured and the fNIRS cap was fitted to align with vertex. Due to individual differences in head shape around the parietal cortex, it is essential to precisely localise each optode for every participant. After positioning the fNIRS cap, we used the 3D motion tracking system from Polhemus (Colchester, Vermont, USA) to record the coordinates of each optode and key anatomical landmarks, including the nasion, inion, right auricular, left auricular, and vertex. This process was conducted using the AstLiteLaunch software and a custom MATLAB script (version R2021b, MathWorks, Inc., Natick, MA, USA). The Polhemus recordings were normalised with custom MATLAB scripts and SPM12 (Tak et al., 2016) to obtain MNI coordinates for each optode. 3D plots of these MNI coordinates were generated for each participant and visually inspected to assess the spatial distribution and consistency, ensuring accurate registration of the Polhemus data. For participants whose MNI optode locations appeared incorrect during visual inspection (*n*= 13), likely due to magnetic interference or participant movement during digitisation, the average MNI coordinates were assigned to all channels. To further validate optode registration, we performed a distance check between the two optodes defining each channel, confirming that distances fell within the expected range of 2.5 to 3.5 cm (corresponding to the 3 cm distance between optodes in our cap configuration). Channels with distances outside this range were flagged (*M*= 2, range: 0–8). For flagged channels, MNI coordinates were replaced with the average MNI coordinates computed from channels with valid distances. We also assessed whether more than 50% of channels for any participant were flagged, considering the replacement of all their locations with average MNI coordinates; however, none met this threshold. Following these corrections, accurate MNI recordings were used to calculate the midpoint between the two optodes for each channel, aligning with our head configuration for each participant.Figure 4Billustrates data for 10 channels on the left hemisphere and 10 channels on the right hemisphere across all participants, with each colour representing corresponding channel positions across the two hemispheres.

#### 2.5.2 Signal preprocessing

First, the raw voltage data of the LightNIRS device from all participants were converted into a Homer-compatible format using the HomER3 software package (Huppert et al., 2009) with a custom MATLAB script. Then, a series of quality checks were performed using custom MATLAB scripts following the procedure outlined byPinti et al. (2019). Specifically, channels were excluded if the PSD lacked clear heartbeat oscillation around 1.2 Hz, showed multiple flat horizontal lines (indicating saturation), or if the intensity time series showed large motor artefacts or appeared flat (indicating signal loss). Out of 1,200 valid channels across all participants, 1,162 survived this thresholding process. At this stage, one participant was excluded due to poor signal quality (as the majority of their channels exhibited flat or near-flat signals), and two others were excluded due to technical errors during data acquisition, reducing the total number of participants to 60. After these exclusions, each channel retained data from an average of 58 participants (ranging from 54 to 60). Using the HomER3 toolbox, the raw intensity data were converted to changes in optical density (OD) to normalise the light intensity measurements using the hmrR_Intensity2OD function. Motion artefacts were corrected using a wavelet-based method with an interquartile range threshold of 1.5, applied through the hmrR_MotionCorrectWavelet function, in line with previous face-to-face social interaction paradigms (e.g.,Pinti et al., 2021;Su et al., 2020). We also filtered the OD data using the hmrR_BandpassFilt function with a 4th-order Butterworth band-pass filter (0.01–0.4 Hz), removing low-frequency drifts (below 0.01 Hz) and high-frequency noise (above 0.4 Hz) consistent with established practices in fNIRS research (e.g.,Pinti et al., 2018,^2019^). Subsequently, pre-processed OD data were converted to changes in oxyhaemoglobin (HbO_2_) and deoxyhaemoglobin (HbR) concentrations using the modified Beer–Lambert law (Delpy et al., 1988) via the hmrR_OD2Conc function. Given the wavelengths used in this study, the differential path length factor (DPF) was approximated as six for both channels (van der Zee et al., 1992). Finally, the Correlation-Based Signal Improvement (CBSI) method (Cui et al., 2010) was applied to combine HbO_2_and HbR signals into an activation signal. Finally, data from the 20 channels were allocated to 12 ROIs using the method described in Section 3 of theSupplementary Materials. This process enabled the transition from overlapping channel locations (Fig. 4B) to discrete ROIs (Fig. 4C). The centres of each ROI are summarised inTable 2. High-quality ECG data were available for 40 of 60 participants. For each trial, the mean HR was calculated for the whole trial and these data were included as a regressor in the design matrix. Further analyses of heart rate data will be presented in a different paper.

**Table 2.** MNI coordinates for the 12 ROIs included in this analysis.

Region	Laterality	X	Y	Z	*N*
**STS**	R	57	-61	11	49
**TPJ**	R	51	-51	20	45
**AG**	R	53	-41	49	55
**IPL**	R	49	-56	35	51
**SMG**	R	56	-36	32	50
**IPS**	R	37	-52	54	55
**STS**	L	-54	-67	12	47
**TPJ**	L	-50	-52	21	49
**AG**	L	-51	-44	48	52
**IPL**	L	-46	-63	35	56
**SMG**	L	-53	-44	32	49
**IPS**	L	-35	-58	54	58

STS = posterior superior temporal sulcus; TPJ = temporo-parietal junction; AG = angular gyrus; IPL = inferior parietal lobule; SMG = supramarginal gyrus; IPS = intraparietal sulcus; L = left; R = right. N indicates the number of participants contributing data in this ROI.

### 2.6 fNIRS data analysis

The CBSI-based activation signals were analysed using a General Linear Model (Friston et al., 1994) implemented in MATLAB (version R2021b) with SPM12 (Ashburner et al., 2021) and NIRS-SPM (Tak & Ye, 2014). For each participant, a design matrix was constructed with six regressors tied to timestamps from the experimental procedure (seeSection 2.2.1). Three regressors were included for the BeMim block: MC (BeMim-Congruent), MI (BeMim-Incongruent), and MB (BeMim baseline), and three regressors for the No-BeMim block: NC (No-BeMim-Congruent), NI (No-BeMim-Incongruent), and NB (No-BeMim baseline). Specifically, MC, MI, NC, and NI were modelled from the “Participant two?” command until “Trial end”, capturing only the period when the confederate’s response could either mimic or not mimic the participant. Meanwhile, MB and NB were modelled from “Which picture do you like?” to “Participant two?” across all trial types (MC, MI, MB, NC, NI, NB). This approach ensures that the baseline primarily reflects the participant’s own processes: examining the images, deciding, and making their choice. Additionally, heart rate was included in the GLM as a covariate to account for potential physiological confounds. From the fitted GLM, beta values were estimated for each fNIRS channel and each regressor. For each participant, we calculated the average beta values from all channels allocated to each ROI, based on the ROI allocations described above. This process resulted in a single beta value for each condition and each ROI per participant.

Statistical tests on the beta values were conducted both within and between the Choice and Motor groups, focusing on four primary contrasts. First, the main effect of the BeMim condition was tested by comparing congruent and incongruent trials in the BeMim block with those in the No-BeMim block as (MC + MI) > (NC + NI). Second, the simple effect of being mimicked was examined with (MC > NC), including only the congruent trials in the BeMim and No-BeMim blocks, as these trials made up the majority in each block and the confederate’s behaviour was predictable. Third, an interaction effect specific to mimicry trials was examined using (MC + NI) > (NC + MI). That is, any brain region that is active to a trial where the participant is mimicked (regardless of the block) should be visible in this contrast. Finally, the main effect of congruency was tested by comparing congruent and incongruent trials across BeMim and No-BeMim conditions as (MC + NC) > (MI + NI). For each contrast, paired t‐tests were performed within the 12 ROIs to compare brain activation in the Choice and Motor groups, and independent t‐tests were used to compare activation between these groups. Brain regions that showed significant results for the main effect of the BeMim condition, the interaction effect, or the main effect of congruency were further examined for additional simple effects, as detailed in the Results section. As the baseline differs from the BeMim and No-BeMim regressors in terms of the participants’ actions, contrasts between BeMim and No-BeMim and baseline are not included in the primary analyses; these contrasts are reported in theSupplementary Materials(Section 7). We also conducted a supplementary investigation linking the BeMim effect to warmth ratings of the confederate that can be found in theSupplementary Materials(Section 8).

Furthermore, an exploratory analysis was conducted to examine how participants’ awareness of the study’s goal influenced the fNIRS BeMim effect, including both between‐group comparisons (Choice vs. Motor) and within‐group analyses. Participants were coded as 1 if they were aware of the study’s goal and/or recognised that they interacted with confederates, and as 0 otherwise. Beta values for the BeMim effect contrast (MC > NC) were first extracted from MATLAB for each of the 12 ROIs and then imported into R. Beta values were standardised as z-scores either across both groups or within each group to facilitate interpretation. Two‐way ANOVAs were then conducted across all 12 ROIs to evaluate the effect of awareness levels in the Choice and Motor groups. Subsequently, Welch two‐sample t‐tests were performed separately within each group to compare the two awareness levels. Each analysis focused on a specific ROI to determine how awareness influenced brain activity, as measured by fNIRS.

## 3 Results

### 3.1 Behavioural results for ratings after each block

To investigate how group allocation (Choice vs. Motor) and experimental condition (BeMim vs. No-BeMim) influence participants’ ratings of the warmth of the confederate, a linear mixed-effects model was constructed as follows: perceived warmth ~ group × condition + (1 | participant ID) + (1 | question). In this framework, group was a between-subjects factor, while condition served as a within-subjects factor. Interaction terms were included to assess the combined effects of these variables. Participant ID and question (e.g., “Do you think [confederate] is a warm person?”) were modelled as random effects to account for variability across individuals and specific questions. The analysis of perceived warmth revealed a significant main effect of group (*β*= 0.46,*p*= .008), indicating that the Motor group gave higher warmth ratings than the Choice group. There was also a significant main effect of condition (*β*= 0.78,*p*< .001), showing that the BeMim condition led to higher warmth ratings than the No-BeMim condition. Critically, the interaction between group and condition (*β*= -0.72,*p*< .001) showed that the BeMim condition had a larger impact on perceived warmth in the Choice group than in the Motor group.

**Fig. 5.**
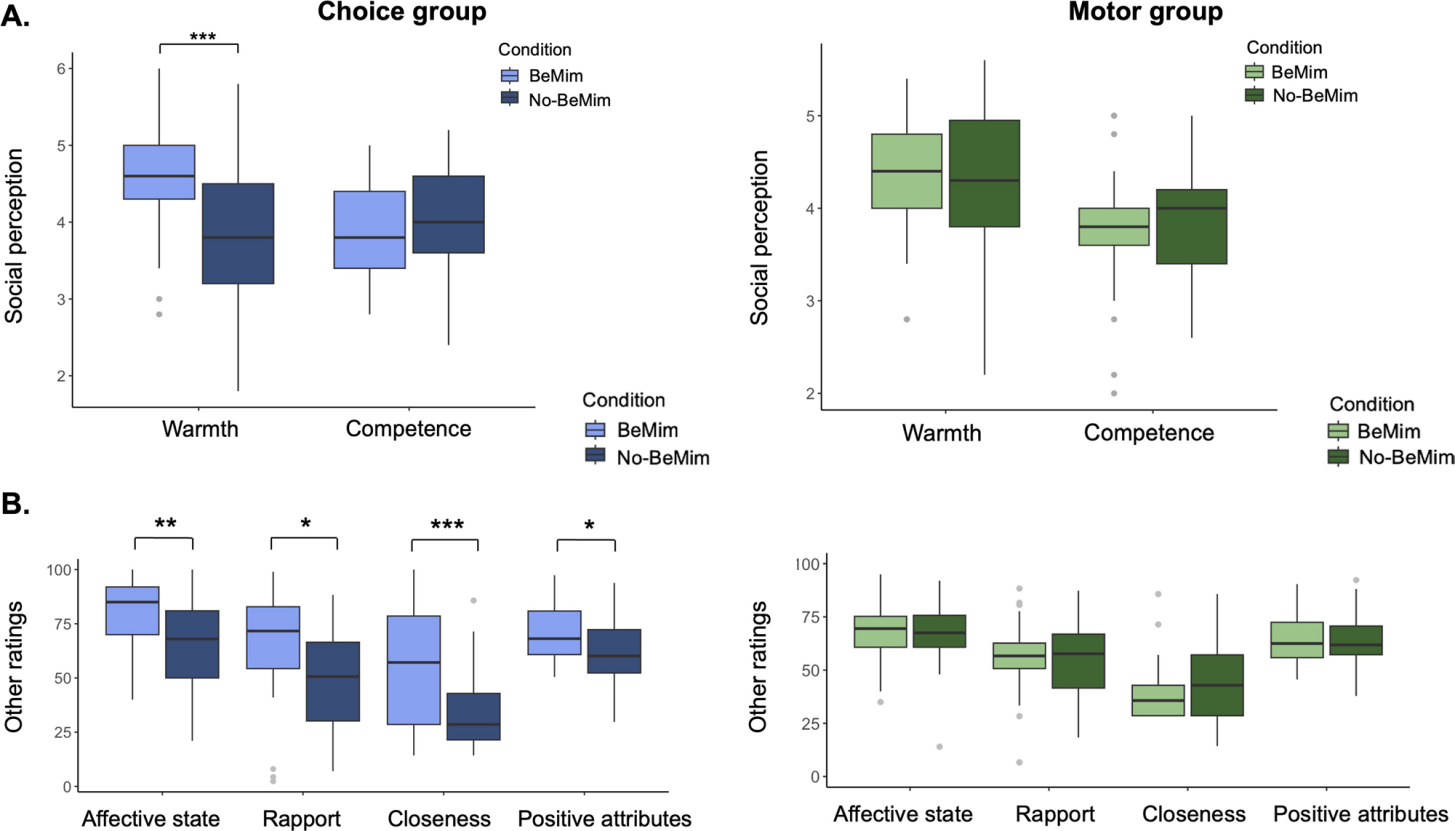
Rating results. (A) Perceived warmth and competence of confederates in the Choice and Motor groups. (B) Additional ratings: perceived affective state, rapport, closeness, and positive attributes scores for Choice and Motor groups. Significant condition effects are indicated with asterisks (*p< .05, **p< .01, ***p< .001).

To follow up on this result, we split the data into the two groups and ran two separate linear mixed-effects models as follows: perceived warmth ~ condition + (1 | participant ID) + (1 | question). The analysis of perceived warmth for the Choice group showed a significant BeMim effect (*β*= 0.78,*p*< .001), indicating that participants in the BeMim condition reported significantly higher warmth scores than those in the No-BeMim condition. In contrast, for the Motor group, the perceived warmth did not differ between BeMim and No-BeMim conditions (*β*= 0.06,*p*= .582). These findings highlight that being mimicked by a confederate increases the participant’s judgement of the warmth of the confederate only in the Choice group (Fig. 5A).

The pattern we find here, with a robust group by condition interaction, was also seen in most of our other rating scale measures as summarised inTable 3. That is, ratings of rapport, closeness, and current affective state all showed an interaction driven by a simple effect of BeMim > No-BeMim in the Choice group only, with a marginal effect in the same direction for the ratings of perceived positive attributes. In contrast, the competence ratings did not change between the BeMim and No-BeMim conditions or show effects of group or any interactions.Figure 5Billustrates the additional ratings scores for Choice and Motor groups. Detailed results for social perception measures (Supplementary Table S2) and additional rating measures (Section 5) are provided in theSupplementary Materials. Additionally, the results of models including the confederate’s name as a factor, used to assess the potential influence of pre-existing biases, are detailed in Section 4.1 of theSupplementary Materials.

**Table 3.** Summary of results for all rating scales.

Rating scale	Main BeMim effect	Main group effect	Interaction	Simple effect within Choice group
Current affective state	***p*** **<** **.001**	*p* = .850	***p*** **=** **.015**	***p*** ***=*** **.003**
Perceived warmth	***p*** **<** **.001**	***p*** **=** **.008**	***p*** **<** **.001**	***p*** **<** **.001**
Perceived competence	*p* = .601	*p* = .423	*p* = .927	*p* = .597
Perceived rapport	***p*** ***=*** **.002**	*p* = .152	***p*** **=** **.013**	***p*** ***=*** **.010**
Perceived closeness	***p*** **<** **.001**	*p* = .233	***p*** **=** **.001**	***p*** **<** **.001**
Perceived positive attributes	*p* = .786	*p* = .842	*p* = .060	***p*** **=** **.027**

Bold values indicate statistically significant*p*-values.

### 3.2 Behavioural results for tasks after both blocks

**Fig. 6.**
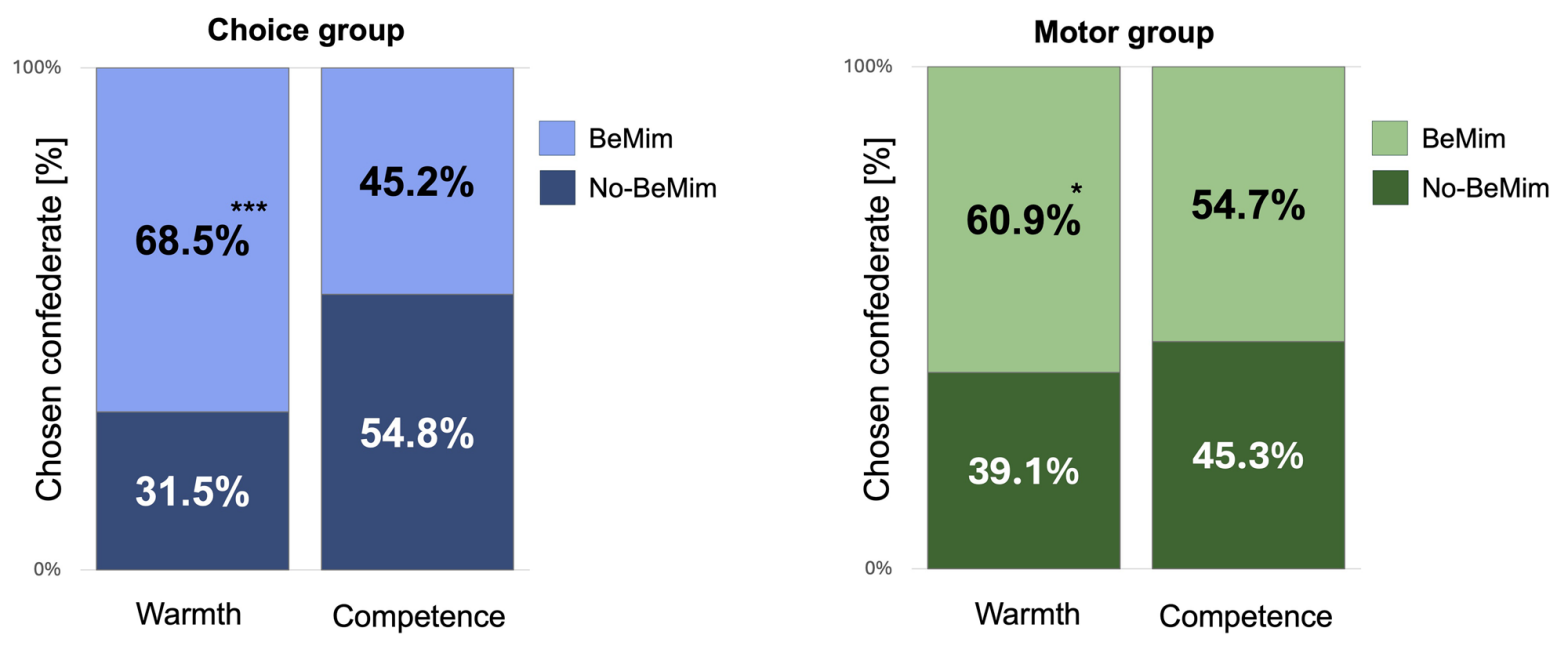
Results from measures applied after both conditions. Behavioural intentions regarding the preferred confederate in warmth and competence-related scenarios, separately for the Choice and Motor groups. Significant preferences for the BeMim confederate over the No-BeMim confederate are indicated with asterisks: *p< .05, ***p< .001.

After participants completed both blocks of trials, we could assess which of the two confederates they prefer using a set of behavioural intentions questions related to warmth and a second set of questions related to competence. To analyse these, we used four separate chi-squared tests of independence. These tests assessed whether participants in each group preferred the BeMim confederate over the No-BeMim confederate in warmth and competence-related scenarios. The results indicated that the Choice group showed a significant preference for the BeMim confederate in warmth-related scenarios,*χ²*(1,*N*= 124) = 17.07,*p*< .001, but not in the competence-related scenarios,*χ²*(1,*N*= 124) = 1.16,*p*= .281. Similarly, the Motor group also preferred the BeMim confederate in warmth-related scenarios,*χ²*(1,*N*= 128) = 6.13,*p*= .013, but showed no significant preference in competence-related scenarios,*χ²*(1,*N*= 128) = 1.13,*p*= .289. Note that this result contrasts with the earlier measures, because here there is a positive impact of mimicry in the Motor group on a warmth-related measure, whereas the rating scales found a null result in the Motor group.Figure 6displays the percentage of participants’ choices for the BeMim and No-BeMim confederates across both groups. The results of the Maze Game analysis are presented in theSupplementary Materials(Section 6).

### 3.3 fNIRS results for Choice and Motor groups

**Fig. 7.**
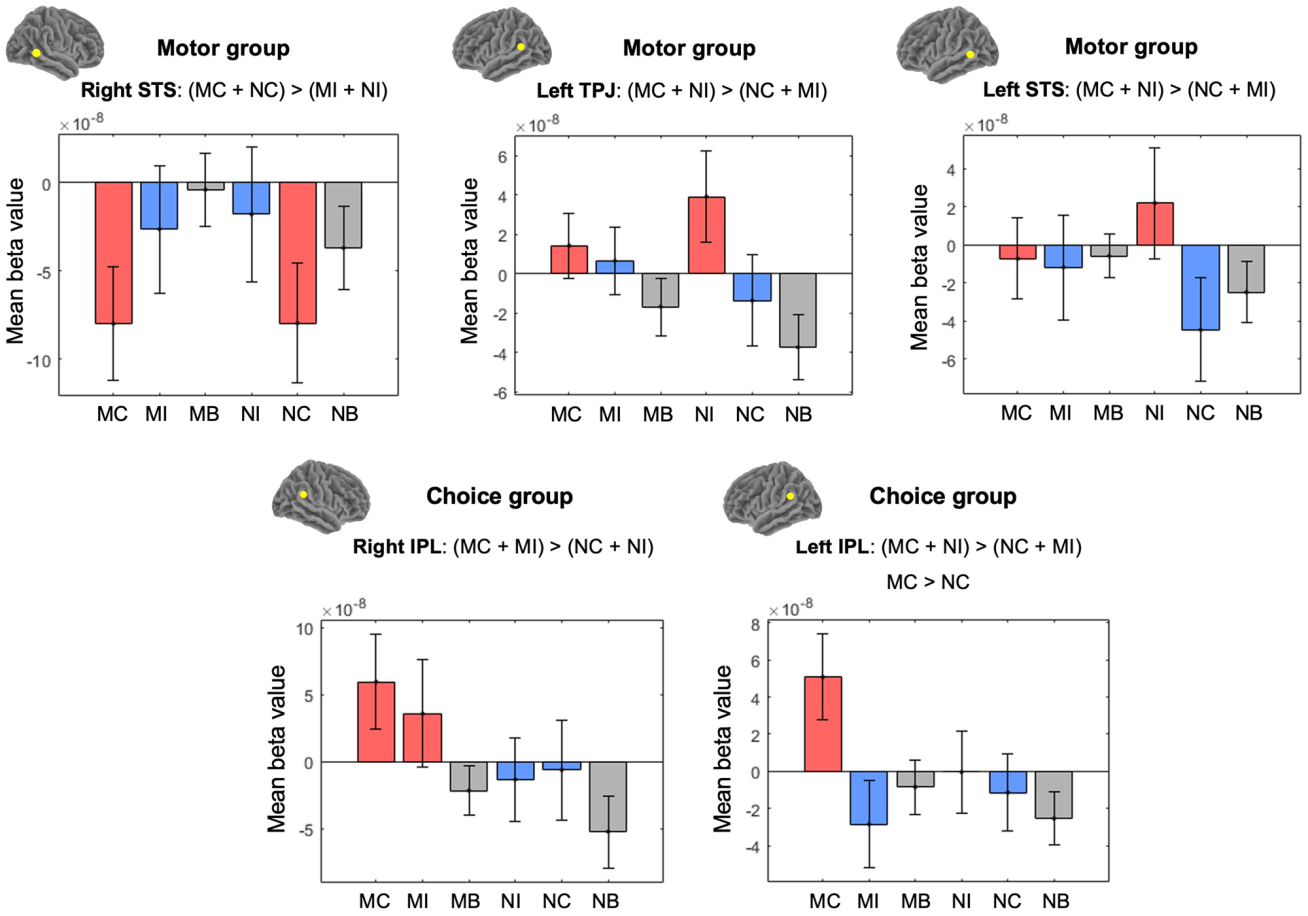
fNIRS results for Choice and Motor mimicry groups. MC = BeMim-Congruent trials, NC = No-BeMim-Congruent trials, NI = No-BeMim-Incongruent trials, MI = BeMim-Incongruent trials.

In the Motor group, a significant difference emerged between congruent (MC + NC) and incongruent (MI + NI) trials in the right STS, showing a smaller decrease from zero in the incongruent trials,*t*(24) = -2.91,*p *= .008, Cohen’s*d*= .58 (Fig. 7). A follow-up evaluation of simple effects indicated decreased haemodynamic activity during NC compared with NI in the right STS,*t*(24) = -2.15,*p*= .042, Cohen’s*d*= .43.

Furthermore, both the left STS and the left TPJ showed a significant interaction effect for contrast MC + NI > NC + MI, identifying regions more active in any mimicry trial relative to any non-mimicry trial (irrespective of block). The left TPJ,*t*(26) = 2.31,*p*= .029, Cohen’s*d*= .44, and the left STS,*t*(22) = 2.19,*p*= .039, Cohen’s*d*= .46, both showed heightened activity during mimicry trials (Fig. 7). A subsequent simple-effects analysis revealed increased haemodynamic activity during NI compared with NC in the left STS,*t*(22) = 2.25,*p *= .035, Cohen’s*d*= .47. No significant results emerged in the Motor group for the main effect of the BeMim condition or the simple effect of being mimicked.

In the Choice group, a significant main effect was observed for the BeMim condition, with higher haemodynamic activity during the BeMim block (MC + MI) than during the No-BeMim block (NC + NI),*t*(25) = 2.47,*p*= .020, Cohen’s*d*= .49. In addition, the simple effect of being mimicked revealed greater haemodynamic changes in the left IPL for BeMim-Congruent trials (MC) than the No-BeMim-Congruent trials (NC),*t*(29) = 2.25,*p*= .033, Cohen’s*d*= .41 (Fig. 7).

Analysis of the interaction effect MC + NI > NC + MI revealed a positive effect in the Choice group, with greater activity in the left IPL during mimicry than non-mimicry trials,*t*(29) = 2.62,*p *= .014, Cohen’s*d*= .48 (Fig. 7). A follow-up simple effects analysis indicated increased haemodynamic activity during MC compared with MI in the left IPL,*t*(29) = 3.26,*p*= .003, Cohen’s*d*= .60. Note this effect was significant after FDR correction (*p*= .034). Tables of statistics for all ROIs across all contrasts in the Motor and Choice groups (Sections 9A and 9B), along with multiple comparisons of the reported effects and recommended sample sizes for future studies (Section 10), are provided in theSupplementary Materials.

### 3.4 fNIRS results for Choice versus Motor groups

**Fig. 8.**
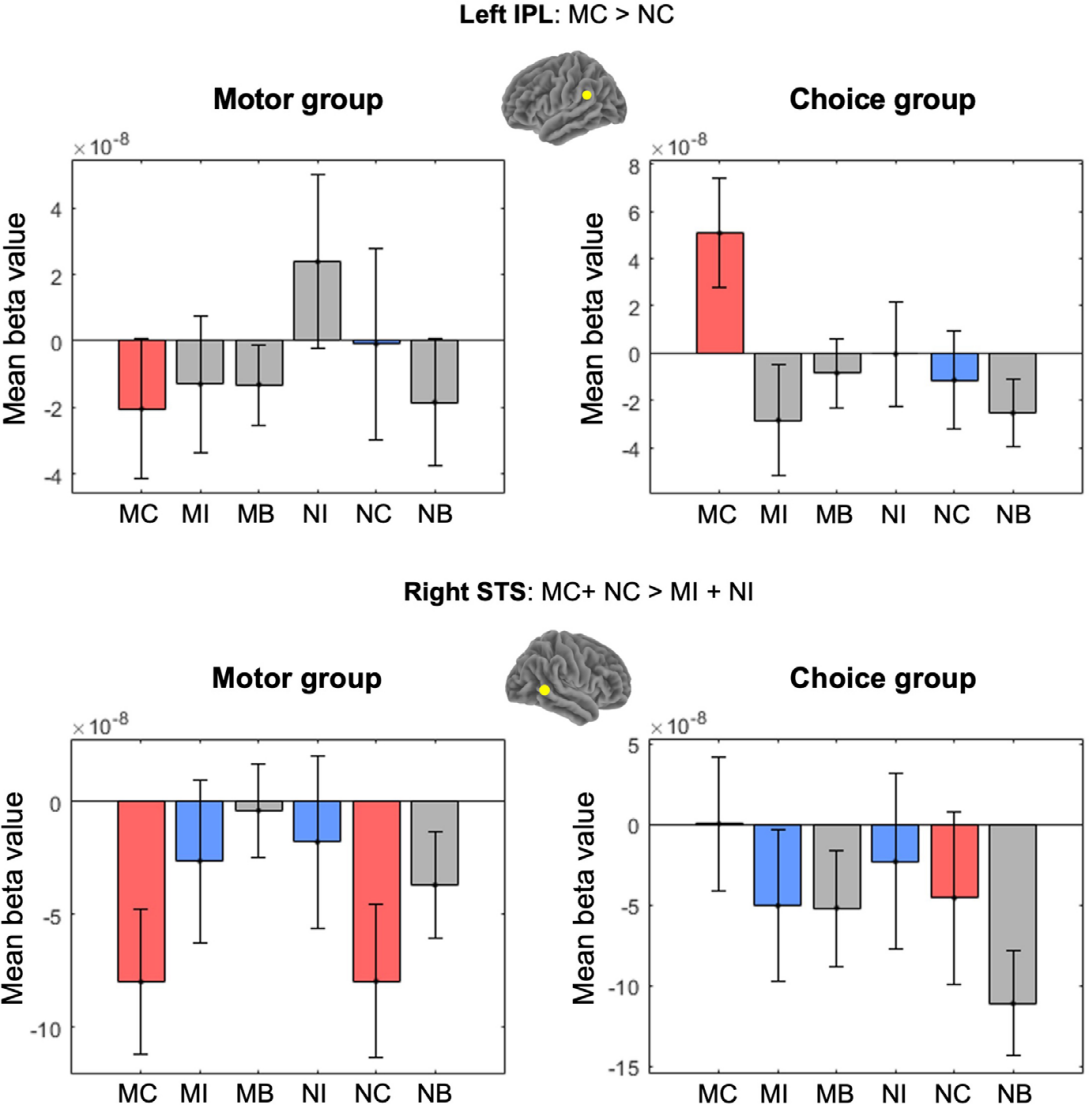
fNIRS results for Choice versus Motor mimicry groups. MC = BeMim-Congruent trials, NC = No-BeMim-Congruent trials, NI = No-BeMim-Incongruent trials, MI = BeMim-Incongruent trials.

A comparison between the Motor and Choice groups for the effect of being mimicked revealed a significant group difference in the left IPL. That is, the contrast (Motor MC – Motor NC) was significantly smaller than the contrast (Choice MC – Choice NC),*t*(56) = -2.05,*p *= .045, Cohen’s*d*= .54 (Fig. 8). This finding suggests that the enhanced response to mimicry congruent trials in the left IPL is specific to the Choice group.

Furthermore, a significant group difference was found in the main effect of congruency for the right STS, with the Motor group showing a significantly smaller difference between congruent and incongruent trials (regardless of the condition) compared with the Choice group,*t*(47) = -2.11,*p *= .040, Cohen’s*d*= .60 (Fig. 8). This indicates that the right STS in the Motor group is more strongly activated by incongruent trials than in the Choice group. Follow-up analyses of the simple between‐groups congruency effects confirmed that the reduction in the haemodynamic activity during BeMim‐Congruent (MC) relative to BeMim‐Incongruent (MI) trials in the right STS was more pronounced in the Motor group,*t*(47) = -2.33,*p*= .024, Cohen’s*d*= .67.

In addition, a significant group difference was found in the left IPL,*t*(56) = -2.69,*p*= .009, Cohen’s*d*= .71, indicating that the MC > MI contrast was larger in the Choice group than in the Motor group. Taken together, these results suggest that while the right STS shows a robust sensitivity to trial incongruency in the Motor group, the enhanced response to mimicry congruent trials in the left IPL is specific to the Choice group. No other between‐groups contrasts yielded significant results. Tables of statistics for all ROIs across all contrasts comparing the Motor and Choice groups are provided in Section 9C of theSupplementary Materials.

### 3.5 Influence of study goal awareness on fNIRS activation

An exploratory logistic regression was used to determine whether participants’ awareness of the study’s goal differed by group (Choice vs. Motor), with “awareness” coded as 1 if participants either accurately guessed the study’s purpose and/or recognised they were interacting with confederates, and 0 otherwise. The results indicated that participants in the Motor group were significantly less likely to be aware than those in the Choice group (*β*= -1.25, SE = 0.59,*z*= -2.14,*p*= .032), with the odds of awareness in the Motor group being approximately 29% of those in the Choice group (OR = 0.29).

To examine how awareness differed across groups in the fNIRS data, we conducted an exploratory 2‐way ANOVA across 12 ROIs for the BeMim effect contrast (MC > NC). In the right IPS, there was a main effect of awareness,*F*(1, 52) = 10.68, FDR‐corrected*p*= .003, indicating that differing awareness levels were associated with distinct mean activation values. A significant interaction between awareness and group was observed,*F*(1, 52) = 6.19, uncorrected*p*= .016; however, this effect did not remain significant after FDR correction (*p*= .109), suggesting only a trend for an awareness–group interaction in the right IPS. Subsequent Welch two‐sample t‐tests were performed to investigate these findings within each group. In the Choice group, participants who correctly guessed the study’s goal and/or recognised the confederates had significantly higher right IPS activation than those who did not,*t*(19) = 3.60, FDR‐corrected*p*= .023, Cohen’s*d*= 1.47. In contrast, in the Motor group, no difference in right IPS activation emerged between aware and unaware participants,*t*(10) = 0.23, FDR‐corrected*p*= .998, Cohen’s*d*= .01 (seeFig. 9).

**Fig. 9.**
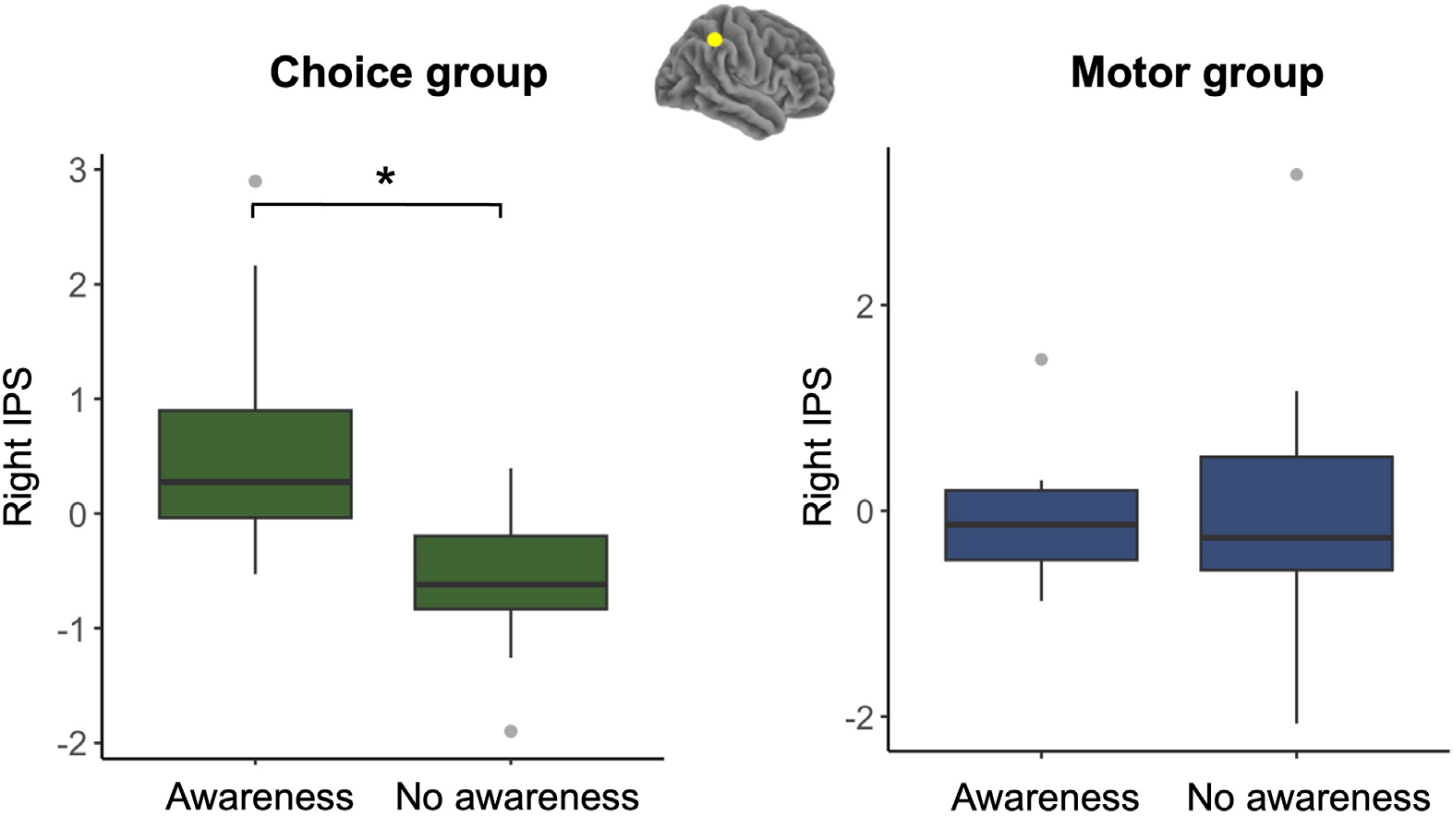
Right IPS activation by study‐goal awareness in the Choice and Motor mimicry groups. An asterisk (*) indicates a significant difference (*p*< .05).

## 4 Discussion

The aim of this study was to explore the behavioural, cognitive, and neural effects of being mimicked in terms of one’s choices and motor movements. Participants in the Choice and Motor groups engaged with both mimicking and non-mimicking confederates. During these interactions, brain activity in the temporal and parietal regions was recorded using fNIRS, and BeMim effects were assessed through multiple measures. This is the first study of its kind to measure brain activity when being implicitly mimicked in real time during in-person social interaction. As predicted, the behavioural results confirmed that choice mimicry had a stronger affiliative effect than motor mimicry. Motor BeMim also activated the left posterior superior temporal sulcus and temporo-parietal junction, whereas Choice BeMim was linked to activity in the bilateral inferior parietal lobule. Finally, awareness of the study’s goal increased the right intraparietal sulcus activity in the Choice group but did not do so in the Motor group. The implications of these findings for future research are discussed below.

### 4.1 Effects of motor mimicry

The first notable finding in our data is the detailed behavioural analyses of how being mimicked impacts on ratings related to affiliation and warmth as well as measures of behavioural intentions. The claim that motor mimicry acts as a social glue between people is widely believed (Chartrand & Bargh, 1999;Dijksterhuis, 2005;Lakin et al., 2003), though effects may be fragile (Hale & Hamilton, 2016b). Here, we find that being mimicked in terms of motor actions did not cause participants to like the confederate in the majority of our measures including perceived warmth, rapport, and closeness. However, a positive effect of Motor BeMim was found in the warmth-related behavioural intentions measures where participants were forced to select which of two confederates they preferred. This task may capture more subtle effects than the rating scales. The very weak liking effect induced by Motor BeMim is consistent with other studies which find null or ambiguous results when evaluating the consequences of mimicry (Hale & Hamilton, 2016a;Maddux et al., 2008, Study 2;Majka et al., 2020;Rauchbauer et al., 2020;van Swol, 2003). Our results suggest that detecting such subtle effects may depend on the sensitivity of the measures used, with binary choices proving to be more effective.

A strong interpretation of our findings would challenge the hypothesis that Motor BeMim acts as a “social glue,” or that imitating someone’s actions robustly enhances affiliation (Kavanagh & Winkielman, 2016;Lakin et al., 2003). However, our BeMim task which created discreet trials with matching or non-matching arm movements is quite different to the more natural gesture-based mimicry studied byChartrand and Bargh (1999). More evaluation of the impact of natural BeMim on affiliation with large sample sizes and pre-registration of results would be valuable to give a better evaluation of the social glue hypothesis.

Contrary to our initial expectations, the neural data for the Motor group indicated engagement of the social brain network. Specifically, mimicry trials (MC and NI), compared with no-mimicry trials (NC and MI), elicited stronger activation in the left STS and left TPJ, areas linked to distinguishing self from other (Brass et al., 2009;Decety et al., 2002) and perspective taking (Carrington & Bailey, 2009;Jääskeläinen & Kosonogov, 2023;Ruby & Decety, 2001). It is worth noting that in the left STS, this effect appeared to be driven by the difference between NC and NI trials under the no-mimicry condition. These findings build on earlier research implicating the STS and TPJ in overt Motor BeMim (Brass et al., 2009;Miyata et al., 2021), emphasising their importance in implicit Motor BeMim.

We also observed increased activation in the right STS for incongruent (MI and NI) compared with congruent (MC and NC) trials across both conditions, indicating that the right STS is sensitive to actions that differ from a general pattern. Moreover, the group comparison analysis confirmed that this effect was stronger for the Motor group than for the Choice group. This result aligns with studies reporting enhanced right STS activity when expected and observed actions do not match (Pelphrey et al., 2004) or when an action appears inconsistent with presumed intentions (Vander Wyk et al., 2009). Our results extend this to the context of being mimicked in motor movements, suggesting that the right STS can detect inconsistent actions that do not align with a person’s usual behaviour.

We did not confirm our initial hypothesis that Motor BeMim would engage the MNS, which was based on the earlier work that assessed Motor BeMim alongside mimicry production (Brass et al., 2009;Miyata et al., 2021). AlthoughDecety et al. (2002)reported increased IPL activation during explicit Motor BeMim compared with Motor No-BeMim, we did not replicate this result under implicit Motor BeMim conditions. Similarly,Miyata et al. (2021)observed more IPL activity when participants mimicked deliberate facial movements relative to a baseline, whereas our main contrasts centred on mimicry versus no-mimicry trials. It is worth noting that both of these previous studies were conducted in relatively less socially engaging settings inside an fMRI scanner. In contrast, our research investigated non-explicit, in-person mimicry of hand actions, suggesting that the MNS may not play a significant role in implicit, face-to-face Motor BeMim.

Lastly, in our exploratory analysis, we did not find evidence that awareness of the study’s purpose modulated neural activation in the temporal or parietal regions in the Motor group. Although previous research suggests that awareness of motor mimicry can influence participants’ responses on a behavioural level (Chartrand & Bargh, 1999;Kulesza et al., 2016), we observed no impact on brain activity. Given the limited research in this area, and to our knowledge, the first report of a null effect of awareness on neural responses to motor mimicry, further investigation is needed. As this outcome is exploratory and drawn from a small group of participants who recognised the study’s goal (only 6 out of 30), more research is required to establish the role of awareness in shaping neural responses to mimicry.

### 4.2 Effects of choice mimicry

The Choice BeMim results confirmed the hypothesis that participants liked confederates who mimicked their art preferences more than those who did not. This affiliative effect was reflected across various measures, indicating strong effect sizes in perceived warmth, affective state, rapport, closeness, positive attributes, and warmth-related behavioural intentions. This aligns withFarmer et al. (2019), who found that participants preferentially liked agents who mimicked their choices and perceived them as more similar compared with agents who did not mimic. This study provides evidence that copying someone’s choices in a live social context may be similar to homophily (Louch, 2000;McPherson et al., 2001). While Choice BeMim focuses on the influence of one person on another through the act of mimicking preferences, choice homophily relies on identifying common traits based on limited information and does not necessitate direct interaction. Despite those differences, both mechanisms may be interconnected, leading to increased affiliation, as similarity-attraction theory suggests that people are attracted to those who are similar to them (Byrne, 1961;Launay & Dunbar, 2015;McPherson et al., 2001).

Turning to the neural data, we observed increased activation in the left IPL during mimicry trials (MC and NI) compared with no-mimicry trials (NC and MI) in the Choice group. It is important to note that this result was primarily driven by the difference between mimicry-congruent (MC) trials and mimicry-incongruent (MI) trials, which remained significant after the FDR correction. We also found greater left IPL activity in mimicry-congruent (MC) than in no-mimicry-congruent (NC) trials, and a between-group comparison confirmed that the enhanced response to MC over MI was stronger in the Choice group than in the Motor group. Taken together, these findings imply that the left IPL may be specifically activated when the alignment of choices is congruent. In contrast, we found greater activation in the right IPL under the BeMim condition (MC and MI) than the No-BeMim condition (NC and NI), suggesting that the right IPL may be particularly involved in monitoring the mimicker’s behaviour, irrespective of whether the choices are congruent.

Collectively, these activations imply that the IPL may track the mimicker’s intention behind choices, in line with research linking the bilateral IPL to recognising the goals of observed actions (Grafton & Hamilton, 2007;Patri et al., 2020). Specifically, both the left and right IPL showed strong activation during mimicry-congruent (MC) trials, where the participant’s choices were mimicked by the mimicker, but not during any trials of the non-mimicry block (NI and NC). However, whereas the left IPL response was specific to these congruent (MC) trials, the right IPL was also active during incongruent (MI) trials by the same mimicker, suggesting a broader role in monitoring the mimicker’s choices. These findings can also be linked to social decision making (Suzuki et al., 2015), which has demonstrated that activity in the bilateral IPL is associated with participants’ inferences about how strongly each choice is maintained by other group members. This suggests that the IPL may encode how participants weigh the confederate’s intentions, reflecting their judgements about how strongly others maintain shared actions.

Thus, contrary to our expectations, we did not find clear evidence of the social brain network involvement during Choice BeMim. Our initial hypotheses about Choice BeMim were derived from research into homophily and the role of social influence in preferences (Campbell-Meiklejohn et al., 2010;Farmer et al., 2019;Suzuki et al., 2015), which did not specifically address the effects of being mimicked in choices. In light of limited literature, we cannot fully explain why our social brain regions were not implicated.

In addition, a larger proportion of participants in the Choice group correctly identified the study’s aim than in the Motor group, and those who recognised it displayed increased activation in the right IPS. This region has been associated with monitoring others’ choices (Suzuki et al., 2015), following other’s gaze (Ramsey et al., 2011), and social evaluation (Cloutier & Gyurovski, 2013;Cloutier et al., 2012). Thus, when participants realised they were being mimicked and/or interacting with confederates, they may have monitored their partner’s behaviour more actively.

### 4.3 Broader interpretations

Consistent with our hypothesis, the behavioural data confirmed that choice mimicry elicited a stronger affiliative response than motor mimicry. There are several potential explanations for this result. First, it may be that people perceive choices to be more personal than actions as studies on homophily found that shared preferences can make people believe that they share common values more widely (Boer et al., 2011). Copying choices also appears more explicit than copying actions, consistent with the higher rate at which participants in the Choice group guessed the study’s aim. Second, while our study’s timing of confederate’s mimicking response should not play a significant role for choices, it may play a more critical role in actions as shown by the literature (Bailenson et al., 2004). In our task, the confederate mimicked motor movements after around 5 seconds delay; a shorter delay might elicit stronger affiliative effects, though the optimal timing remains unclear (Hale & Hamilton, 2016b). Finally, our Motor paradigm’s distinction between BeMim and No-BeMim was subtle compared with BeMim paradigms used previously (Chartrand & Bargh, 1999;Kulesza et al., 2016), in which no-mimicry involves an entirely different body part for both the participant and the confederate. Overall, as this study is the first to directly compare these forms of mimicry and many aspects of Motor BeMim and Choice BeMim remain unknown, the explanations above remain speculative.

At the neural level, we initially expected that Motor BeMim would recruit the MNS, while Choice BeMim would engage social brain networks. Instead, we found the opposite: Choice BeMim involved the bilateral IPL, and Motor BeMim activated the left TPJ and STS. It appears possible that the MNS does not significantly contribute to implicit Motor BeMim, at least under our experimental conditions. Similarly, we did not find evidence of social network involvement in Choice BeMim, although our prediction was inspired by homophily and social influence research on preferences that did not specifically examine Choice BeMim. Future studies are needed to replicate and clarify these effects.

Intriguingly, the incongruency effect we observed in the Motor group for the right STS more closely resembles the findings ofFarmer et al. (2019)on choice homophily, where right STS activity increased when an agent’s choice contradicted their typical preference. In their study, participants viewed an agent’s inconsistent choices on the screen; similarly, other studies (Pelphrey et al., 2004;Vander Wyk et al., 2009) have reported increased right STS activation when participants observed unexpected actions visually. As posterior right STS is linked to interpreting unexpected biological movement and intentions from visual cues (Van Overwalle & Baetens, 2009) and generally responds less to auditory stimuli (Landsiedel & Koldewyn, 2023), it is possible that we did not detect right STS involvement in the Choice group because participants processed choices through auditory commands.

### 4.4 Implications, limitations, and future directions

The current study is the first to shed light on the brain regions potentially involved in experiences of being implicitly mimicked during in-person interactions. Our findings demonstrate that Choice BeMim can activate the MNS, and Motor BeMim can activate social network, and these activations can be captured using fNIRS. Additionally, Choice BeMim fosters greater affiliation than Motor BeMim, with the latter only inducing subtle liking effects as shown by the forced-choice questions. These results have significant implications for mimicry research, challenging previous work stating that individuals who mimic others’ actions are always liked more (Dijksterhuis, 2005;Lakin et al., 2003). We demonstrate that this statement is more applicable to copying choices rather than motor movements.

While the findings on the neural level are intriguing, the exploratory nature of this analysis necessitates further research to replicate the effects and clarify their underlying mechanisms. In this paper, we report data that are uncorrected for multiple comparisons to showcase how future research could address questions of BeMim. In theSupplementary Materials, we detail the recommended sample size for future studies and FDR correction results. Note that the simple effect of congruency in the Choice group does meet FDR. While our study focused on temporoparietal regions, future fNIRS research should expand to include other areas, such as the frontal cortices. fNIRS offers notable advantages for capturing brain activity in realistic social settings, due to its portability and tolerance for participant movement. This technology enables more ecologically valid experimental testing than is typically feasible in scanner-based environments, which is particularly relevant for investigating interactive phenomena, such as mimicry, in real-world or near-real-world contexts.

One possible limitation of our study is the relatively controlled, trial-based task we used in contrast to the more natural mimicry studied previously (Chartrand & Bargh, 1999;Kulesza et al., 2016). Nevertheless, for Motor BeMim, we successfully captured engagement of the social brain network and confirmed the unsuccessful replication of its affiliative function (Hale et al., 2018;Hale & Hamilton, 2016b;Majka et al., 2020;Rauchbauer et al., 2020;van Swol, 2003). We also demonstrated the behavioural effects of Choice BeMim, with high effect sizes, suggesting its involvement in the MNS. We hope that the current paper can provide a starting point for future studies of BeMim effects using a wider range of tasks and contexts.

Overall, our study sheds light on the neural regions involved in social interaction, where participants are being mimicked in their choices and motor actions: an area of study previously overlooked in mimicry research. Through a carefully controlled experimental design, we compared behavioural, cognitive, and neural responses to being mimicked, both across and within choice and motor mimicry forms. By standardising participant actions and minimising verbal communication, we reduced confounding variables. Using both rating tasks and forced-choice measures allowed us to capture subtle effects, thereby providing an in-depth assessment of each type of mimicry. Understanding the interpersonal function of mimicry can be beneficial for developing interventions aimed at neurodivergent individuals, helping them learn how people naturally bond through copying behaviour. This insight can also enhance therapeutic practices where building rapport is an essential part of the process.

### 4.5 Conclusion

In summary, this study demonstrated that the type of behaviour people choose to copy can lead to different outcomes. Copying actions does not seem to be an effective way to induce significant affiliation in others, whereas mimicking more abstract preferences generates a strong liking effect. Our findings indicate that different regions of the brain are responsive to being mimicked, depending on whether the mimicry involves motor movements (social network) or choices (mirror neuron system). These findings have important implications for our understanding of how humans form bonds at both behavioural and neural levels in real life.

## Supplementary Material

Supplementary Material

## Data Availability

Materials, code, and pre-processed data are publicly available on the Open Science Framework (OSF) and can be accessed athttps://osf.io/f6xkp/?view_only=2e6c520f656d4f86916d9d82565d08f4.
