# Supplementary material for: How choice and motor mimicry affect affiliation: An fNIRS study
Source: Imaging Neurosci (Camb). 2025 Jun 16;3:IMAG.a.40. doi: 10.1162/IMAG.a.40 (PMC12319866; doi:10.1162/IMAG.a.40)
Supplement: Supplementary Material [file imag.a.40_supp.pdf]

Supplementary materials

How Choice and Motor Mimicry Affect Affiliation: An fNIRS Study

Paula Wicher, Eva G. Krumhuber, Aiko Murata, Sabina Beganovic, Antonia F. de C. Hamilton

1. Details of all possible trial types

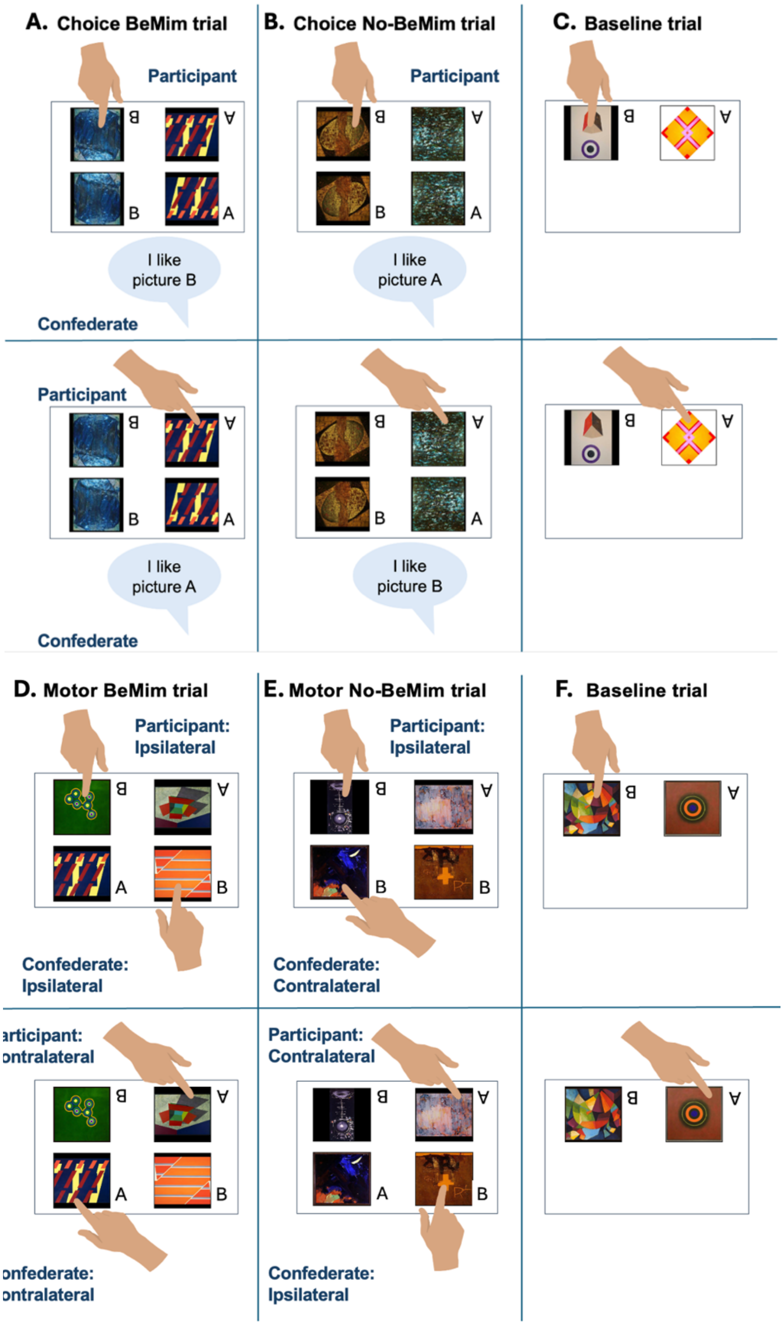

Figure S1. *Trial types in Choice and Motor groups.*

In the Choice group, the participant pointed to their preferred picture, while the confederate pressed a button on a keyboard kept on their lap to generate a voice command indicating their choice. In the **Choice BeMim** trial (1A), both the participant and the confederate chose the same picture. In the **Choice No-BeMim** trial (1B), the confederate selected the opposite image to the participant. In the **baseline** trial (1C), only the participant made a choice.

In the Motor group, both the participant and confederate pointed to the selected image using their right hand. In the **Motor BeMim** trial (1D), they performed the same right-hand action; it could occur in two scenarios depending on the participant's choice. In one scenario (upper 1D), both the participant and the confederate performed an ipsilateral right-hand movement. In the other scenario (bottom 1D), they both performed a contralateral right-hand movement. Thus, the Motor BeMim condition engaged the same motor areas and muscles in a coordinated manner. In the **Motor No-BeMim** trial (1E), they performed different right-hand actions. In the **baseline** trial (1F), only the participant made a choice.

2. **Behavioural measures collected**

Prior to the start of the experiment, participants completed a demographic questionnaire in Gorilla, thereby providing information about their gender, age, education, ethnicity, country of origin, and occupation. To measure individual differences, participants answered two questions about their interest in art ("How interested are you in art?") and art knowledge ("How much do you know about art?") on a scale from 1 (*not interested/knowledgeable at all*) to 6 (*very interested / knowledgeable*). Furthermore, they were asked how many art exhibitions they had visited online or in person in the past two years. The results revealed no major effects.

After each block, participants assessed their current affective state on a scale from 0 (*negative*) to 100 (*positive*) with the question "How did you feel during the art game with [confederate]?". Next, they completed ten items related to the social perception of each confederate using a scale from 1 (*definitely not*) to 6 (*definitely yes*). Five items measured perceived warmth: 1) "Do you think [confederate] is similar to you?", 2) "Do you think [confederate] is a warm person?", 3) "Do you think [confederate] is pleasant to work with?", 4) "Do you think [confederate] is sincere?", 5) "Do you think [confederate] is a good listener?". The other five items assessed perceived competence: 1) "Do you think [confederate] is competent?", 2) "Do you think [confederate] is competitive?", 3) "Do you think [confederate] is intelligent?", 4) "Do you think [confederate] is motivated?", 5) "Do you think [confederate] is an analytical person?". The Cronbach's alpha coefficients for the warmth and competence questions were $\alpha = .83$ and $\alpha = .62$, respectively. To measure perceived rapport with confederates, participants answered three questions on the scale from 0 (*negative*) to 100 (*positive*) (Gratch et al., 2007): 1) "I think [confederate] and I established rapport", 2) "I felt I had a connection with [confederate]", 3) "I think that [confederate] and I understood each other." Participants' perceived closeness with confederates was assessed using the Inclusion of Other in the Self (IOS) scale (Aron et al., 1992). They chose from seven pairs of circles, each representing different levels of overlap to symbolize varying degrees of closeness (1 - *no overlap*, 7 - *most overlap*). Moreover, to assess the type of attributes assigned to the confederate, they indicated the extent to which each confederate possessed the following traits on a scale from 1 (*not at all*) to 100 (*very much*) (Harker & Keltner, 2001): 1) acceptance, 2) generosity, 3) gratitude, 4) sympathy, 5) avoidance, 6) hostility, and 6) distrust.

As part of the ratings after each block, we tested out a task to measure general prosociality. Participants were asked: "If you found a £10 note on the street, how much would you donate to a charity of your choice (the rest you can keep for yourself)?" They selected a charity from a list of London-based options and specified the percentage to donate, ranging from 0% to 100%. Responses showed limited variability, with approximately 47% of participants choosing either the highest value

(100%) or the midpoint (50%), suggesting a tendency toward polarising responses. As a result, this measure was excluded from the primary analysis.

After completing both blocks in each group, participants proceeded to complete additional tasks that force them to choose between the two confederates. Behavioural intentions were measured with four questions related to warmth: 1) "Who would you like to go to the art gallery with?" with response options of [confederate 1 / confederate 2]; 2) "Who would you go for a long walk with?"; 3) "Who would you talk to about a family issue?"; 4) "Who would you ask for drinks in a pub?". Four other questions were related to competence: 1) "Who would you ask to help you with an essay?", 2) "Who would you ask to fix technical problems with your computer?", 3) "Who would you ask to prepare for a job interview?", and 4) "Who would you like to work with on a work project?". Internal consistency of the scales was evaluated using the Kuder-Richardson Formula 20 (KR-20). For the warmth items, the KR-20 was 0.85 for the Choice group and 0.72 for the Motor group. For the competence items, the KR-20 was 0.58 for the Choice group and 0.24 for the Motor group.

After all other tasks, participants completed the Social Interaction Anxiety Scale (Brown et al., 1997), a 20-item measure of social anxiety on a scale from 0 (*not at all*) to 4 (*extremely*), the Interpersonal Reactivity Index (Davis, 2011), a 28-item measure of dispositional empathy on a scale from 1 (*does not describe me well*) to 5 (*describes me very well*), and The Toronto Alexithymia Scale, a 20-item measure of difficulty in identifying and describing emotions on a scale from 0 (*strongly disagree*) to 7 (*strongly agree*) (Bagby et al., 1994). No participants were identified as outliers on these scales, and all were included in the analyses.

3. **Allocation of channels to ROIs**

In our study, a literature-informed approach was used for the ROIs selection. Our aim was to identify candidate brain regions in relevant papers and select ROIs that cover the brain regions where we have available fNIRS data. Initially, we conducted a review of neuroimaging literature related to our study's focus. Key studies were identified on topics such as Choice BeMim (Farmer et al., 2019), production of motor mimicry (Brass et al., 2009; Caspers et al., 2010; Mainieri et al., 2013; Mengotti et al., 2012), self-other distinction (Farrer et al., 2008), social perception (e.g., Cloutier & Gyurovski, 2013), theory of mind (e.g., Chambon et al., 2017), and social learning (e.g., De Felice et al., 2024). After this review process, we plotted MNI coordinates from these studies on a brain surface model alongside our channel data in MATLAB. This method allowed us to visually assess the spatial distribution of our channels relative to the ROIs identified in the literature. Our review revealed a predominant emphasis on right-hemisphere regions. Accordingly, we initially averaged coordinates from right-hemisphere studies and mirrored as well as adjusted them to create ROIs for our left-hemisphere channel data. The studies that were ultimately selected to guide the specification of our ROIs are detailed in Table S1 below. The final 12 ROIs were selected, with 6 for each hemisphere,

including the Intraparietal Sulcus (IPS), Supramarginal Gyrus (SMG), Angular Gyrus (AG), Inferior Parietal Lobule (IPL), Temporoparietal Junction (TPJ), and Superior Temporal Sulcus (STS). Given the exploratory nature of the study, examining multiple ROIs allowed for a broad investigation of the neural mechanisms and potential activation patterns across different brain regions involved in Choice and Motor BeMim.

To allocate channels to specific ROIs, we calculated the distance between each channel and the coordinates of the predefined ROIs. Channels within 12 mm of an ROI's projected location were assigned to the nearest ROI. Channels that were more than 12 mm from any ROI were excluded from the final analysis. As a result, each channel was assigned to a single ROI, though some ROIs received contributions from two channels. On average, 65 data points (range: 54 to 96 data points per ROI) from 51 participants (range: 45 to 58 participants per ROI) were assigned to each ROI from 60 participants.

Table S1. *Studies informing ROI selection in this study.*

Our ROI	Source	MNI in Source	Study focus
rSTS	Farmer et al., 2019	60, -58, 16	Inconsistent choice by other
	Caspers at al., 2010	54, -64, 4	Action imitation
rTPJ	Farmer et al., 2019	44, -42, 20	Inconsistent choice by other
	Brass at al., 2009	52, -54, 21	Congruent > incongruent imitation
	De Felice et al., 2024	58, -56, 18	Social learning
rAG	Farmer et al., 2019	56, -46, 50	Inconsistent choice by other
	Caspers at al., 2010	52, -36, 52	Action imitation
		51, -36, 50	Action observation and action imitation
		54, -36, 52	Hand imitation
	Cloutier & Gyurovski, 2013	54, -51, 39	Self-referential social status judgments
rSMG	Mengotti et al., 2012	56, -36, 32	Non-Specular > Specular mimicry
rIPS	Farrer et al., 2008	44, -50, 60	Perturbed agency
	Caspers at al., 2010	30, -54, 48	Action observation
rIPL	Selected to cover remaining channels		

4. **Social perception of confederates – detailed results**

Table S2. *Social perception ratings of confederates.* The table shows the results for the linear mixed-effects models examining the effects of group (Choice vs Motor) and experimental condition (BeMim vs No-BeMim) on perceived warmth and competence.

**Fixed effects**	***Estimate***	***SE***	***df***	***t-value***	***p-value***
**Ratings related to warmth**					
**Motor vs Choice groups**					
Intercept***	3.83	0.25	6.47	15.53	<.001
Group: Motor **	0.46	0.17	87.54	2.71	.008
Condition: BeMim***	0.78	0.10	562.46	7.45	<.001
Group and condition: Motor vs BeMim***	-0.724	0.15	562.46	-4.93	<.001
**Motor group**					
Intercept***	4.29	0.24	17.60	4.90	<.001
Condition: BeMim	0.06	0.10	283.66	0.55	.582
**Choice group**					
Intercept***	3.83	0.25	7.61	15.37	<.001
Condition: BeMim***	0.78	0.11	274	7.38	<.001
**Ratings related to competence**					
**Motor vs Choice groups**					
Intercept***	3.97	0.31	5.32	12.98	<.001
Group: Motor	-0.13	0.17	94.62	-0.80	.423
Condition: BeMim	-0.06	0.11	562.87	-0.52	.601
Group and condition: Motor vs BeMim	0.01	0.16	562.87	0.09	.927
**Motor group**					
Intercept***	3.83	0.26	14.48	5.50	<.001
Condition: BeMim	-0.04	0.11	284.91	-0.40	.69
**Choice group**					
Intercept***	3.97	0.35	4.90	11.48	<.001
Condition: BeMim	-0.06	0.11	274	-0.53	.597

4.1 **Social perception of confederates – control for pre-existing biases**

To determine whether the observed differences or lack thereof between the BeMim and No BeMim conditions for warmth and competence in the Motor and Choice groups were influenced by pre-existing biases, we employed four linear mixed-effects models. The original models (outlined in section 3.1 in the paper) were modified to include the confederate's stage name as a factor (Anna, Beth, Claire, Diana, Ellie). These models were fitted using the *lmer* function from the lm4 package in R, following the formula: score ~ condition * confederate's name + (1 | participant ID) + (1 | question).

4.1.1 **Motor group**

In the Motor group, regarding perceived competence, the analysis including the confederate's name revealed no significant difference between the BeMim and No BeMim conditions ($\beta = 0.43$, $p = .407$), consistent with the main findings. There were also no significant effects of individual confederates compared to Anna, nor significant interactions between confederates' names and the BeMim condition. For perceived warmth, there was also no significant difference between the BeMim and No BeMim conditions ($\beta = 0.81$, $p = .070$), aligning with the main findings. However, a significant difference was observed for confederate Beth compared to Anna ($\beta = 1.23$, $p = .03$), while comparisons with other confederates were not significant. Additionally, a significant interaction was found between Beth and the BeMim condition ($\beta = -1.22$, $p = .023$). Post hoc pairwise comparisons with Bonferroni corrections revealed no significant differences between the BeMim and No BeMim conditions for each confederate individually.

4.1.2 **Choice group**

In the Choice group, the analysis including the confederate's name as a factor revealed no significant difference between the BeMim and No BeMim conditions in perceived competence ($\beta = -0.60$, $p = .232$), consistent with the main findings. However, significant main effects were observed for confederates Claire ($\beta = -1.04$, $p = .026$), Diana ($\beta = -0.94$, $p = .046$), and Ellie ($\beta = -1.06$, $p = .029$) compared to Anna. There were no significant interactions between confederates' names and the BeMim condition. Post hoc pairwise comparisons with Bonferroni corrections revealed no significant differences between the BeMim and No BeMim conditions for each confederate individually.

For perceived warmth in the Choice group, the analysis showed a significant difference between the BeMim and No BeMim conditions ($\beta = 2.64$, $t(240) = 5.27$, $p < .001$), consistent with the main findings. Significant main effects were found for confederates Beth ($\beta = 1.71$, $p < .001$), Claire ($\beta = 1.33$, $p = .004$), Diana ($\beta = 1.42$, $p = .002$), and Ellie ($\beta = 1.52$, $p = .002$) compared to Anna. Significant interactions were also observed between the BeMim condition and Beth ($\beta = -2.29$, $p < .001$), Claire ($\beta = -1.74$, $p = .005$), Diana ($\beta = -1.58$, $p = .009$), and Ellie ($\beta = -2.38$, $p < .001$). Post hoc pairwise comparisons with Bonferroni corrections indicated significant differences between the BeMim and No BeMim conditions for Anna ($\beta = 2.64$, $p < .001$), Claire ($\beta = 0.91$, $p = .018$), Diana (β

= 1.07, $p = .003$), and no significant differences between BeMim and No-BeMim conditions for Beth ($\beta = 0.35$, $p = .241$) and Ellie ($\beta = 0.26$, $p = .491$). In summary, warmth and competence results were generally unaffected by pre-existing biases related to confederates in the Motor and Choice groups. Including confederate names did not alter the main findings, indicating robust effects across confederates.

4.2 Social perception of confederates - study goal awareness

Information from the debriefing was analysed to assess whether participants' awareness of the study goal influenced their responses in the main experiment. Participants were categorised based on whether they accurately guessed the study's goal and/or recognised that they were interacting with confederates. Those who both correctly identified the study's goal and realised they were interacting with confederates were coded as 1 or 0 otherwise.

To examine the potential impact of participants' awareness of the study goal on warmth-ratings scores, two linear mixed-effects models were conducted using the *lmer* function from the lme4 package in R, separately for the Choice and Motor groups. The model was specified as follows: warmth score ~ awareness * condition + (1 | participant ID). In this analysis, study goal awareness (coded as 1, 0.5, 0) was a between-subjects factor, while experimental condition (BeMim vs No-BeMim) was a within-subjects factor. Participant ID was included as a random effect to account for variability across individuals. For the Choice group, there was no significant effect of awareness ($\beta = 0.27$, $t(49.82) = 0.79$, $p = .435$) nor was there a significant interaction between condition and awareness ($\beta = 0.21$, $t(29) = 0.06$, $p = .955$). Similarly, in the Motor group, there was no significant effect of awareness ($\beta = -0.21$, $t(60) = -0.59$, $p = .557$), and the interaction between awareness and condition was also not significant ($\beta = 0.42$, $t(60) = 0.82$, $p = .419$). Participant's awareness of the study goal did not significantly affect their social perception of confederates.

5. Analysis of additional rating measures

A series of linear mixed-effects models were conducted to assess the impact of group (Choice vs. Motor) and experimental condition (BeMim vs. No-BeMim) on current affective state, rapport, closeness, and confederates' positive attributes. Each model was specified as: response ~ condition * group + (1 | participant ID).

5.2 Current Affective State

The analysis revealed a significant difference in affective state between the BeMim and No-BeMim conditions ($\beta = 13.32$, $p < .001$). Although there was no significant difference between Motor and Choice groups ($\beta = 0.82$, $p = .850$), a significant interaction between condition and group ($\beta = -12.83$, $p = .015$) indicated that the difference in perceived affect between BeMim and No-BeMim was smaller in the Motor group compared to the Choice group. Post-hoc pairwise comparisons with Bonferroni adjustment showed a significant difference in perceived affect between the BeMim and

No-BeMim conditions within the Choice group ($\beta = 13.32$, $t(61) = 3.64$, $p = .003$), as well as a significant difference between BeMim across the Choice and Motor groups, with higher affect scores in the Choice BeMim group ($\beta = 12.00$, $t(112) = 2.78$, $p = .038$).

5.3 Perceived Rapport

Mean rapport scores, calculated from three items per condition, were used as the dependent variable. The analysis demonstrated a significant difference in perceived rapport between the BeMim and No-BeMim conditions ($\beta = 17.08$, $p = .002$). The main effect of group, comparing the Motor and Choice, was not significant ($\beta = 7.67$, $p = .152$), but a significant interaction between condition and group was found ($\beta = -18.50$, $p = .013$), indicating that the perceived rapport effect of BeMim was smaller in the Motor group than in the Choice group. Post hoc pairwise comparisons with Bonferroni adjustment highlighted a significant difference in perceived rapport between BeMim and No-BeMim scores within the Choice group ($\beta = 17.08$, $t(61) = 3.30$, $p = .010$).

5.4 Perceived Closeness

Closeness scores were converted to percentages to align with the other rating scales. A significant difference in perceived closeness was found between the BeMim and No-BeMim conditions ($\beta = 16.59$, $p < .001$). There was no main effect of group when comparing the Motor and Choice groups on perceived closeness ($\beta = 6.48$, $p = .233$). However, a significant interaction between condition and group was observed ($\beta = -19.27$, $p = .001$), showing that the perceived closeness effect of BeMim was smaller in the Motor group than in the Choice group. Post-hoc pairwise comparisons with Bonferroni adjustment further demonstrated a significant difference in perceived closeness between BeMim and No-BeMim in the Choice group ($\beta = 16.59$, $t(61) = 4.08$, $p < .001$).

5.5 Perceived Positive Attributes

Participants rated each confederate on seven attributes (acceptance, generosity, gratitude, sympathy, avoidance, hostility, and distrust), with negative attributes reverse-scored. The mean scores for perceived positive attributes, calculated from these seven items per condition, served as the dependent variable. The analysis demonstrated a significant difference in perceived positive attributes between the BeMim and No-BeMim conditions ($\beta = 7.52$, $p = .004$). There was no main effect of group when comparing the Motor and Choice groups on perceived positive attributes ($\beta = 0.64$, $p = .842$), nor a significant interaction between condition and group ($\beta = -6.84$, $p = .060$). Post-hoc pairwise comparisons with Bonferroni adjustment revealed a significant difference in perceived positive attributes between BeMim and No-BeMim conditions within the Choice group ($\beta = 7.52$, $t(61) = 2.95$, $p = .027$).

Table S3. *Results for additional ratings.* The table shows results of the linear mixed-effects models examining the effects of group (Choice vs Motor), experimental condition (BeMim vs No-BeMim) on perceived current affective state, rapport, closeness, positive attributes of the confederates.

**Fixed effects**	***Estimate***	***SE***	***df***	***t-value***	***p-value***
**Current affective state**					
Intercept***	66.77	3.08	112.39	21.71	<.001
Group: Motor	0.82	4.32	112.39	0.19	.850
Condition: BeMim***	13.32	3.66	61	3.64	<.001
Group and condition: Motor vs BeMim*	-12.82	5.13	61	-2.50	.015
**Perceived rapport**					
Intercept***	48.29	3.79	121.44	12.75	<.001
Group: Motor	7.67	5.31	121.44	1.44	.152
Condition: BeMim**	17.08	5.17	61	3.30	.002
Group and condition: Motor vs BeMim*	-18.50	7.26	61	-2.55	.013
**Perceived closeness**					
Intercept***	35.48	3.85	102	9.22	<.001
Group: Motor	6.48	5.40	102	1.20	.233
Condition: BeMim***	16.59	4.06	61	4.08	<.001
Group and condition: Motor vs BeMim**	-19.27	5.70	61	-3.38	.001
**Perceived positive attributes**					
Intercept***	63.35	2.24	107.04	28.29	<.001
Group: Motor	-0.64	3.19	107.04	-0.20	.842
Condition: BeMim	0.68	2.51	61	0.27	.786
Group and condition: Motor vs BeMim	6.84	3.57	61	1.91	.060

6. **Approach tendencies and inclination to trust in the Maze Game**

As part of the measures after all blocks, social approach tendencies and the inclination to trust the confederate were assessed using a computerized adaptation of the Maze Game (Hale et al., 2018). For this, participants were placed in a virtual maze with two exit doors. They were instructed to leave the maze as quickly as possible and informed that they should seek assistance from one of two confederates to choose the correct exit door. On each trial, the pictures of both confederates A and B

appeared on the screen and participants needed to approach one of them (BeMim or No-BeMim) to get a hint about which door to open (a measure of social approach). After their hint was displayed, participants could follow their advice or reject it (a measure of trust). The Maze Game always included 12 trials regardless of the choices made.

6.1 Approach tendencies in the Maze Game: Choice vs Motor group

To examine the group effect (Choice vs Motor groups) on participants' approach tendencies toward BeMim and No-BeMim confederates, a binomial logistic mixed-effects model was used. The model was specified as follows: approached confederate ~ group + (1 | participant ID) + (1 | trial number). The binary outcome represented whether participants approached the BeMim confederate (coded as 1) or the No-BeMim confederate (coded as 0) across 12 trials. Participant ID and trial number were included as random effects to account for variability. Nine participants were excluded from the analysis due to a technical setup error in the Maze Game. The results revealed no significant difference in approach tendencies between the Motor and Choice groups ($\beta = 0.06$, $p = .790$). Detailed results can be found in Table S4.

6.2 Approach tendencies in the Maze Game: Choice and Motor groups separately

To investigate within-group differences in approach tendencies, two separate Chi-squared tests of independence were conducted for the Choice and Motor groups. These tests assessed whether participants in each group chose to approach the BeMim confederate more often than the No-BeMim confederate during the 12 trials of the Maze Game. In the Choice group, the analysis showed no significant preference for approaching the BeMim over the No-BeMim confederate, $\chi^2(1, N = 324) = 0.20$, $p = .657$. Similarly, the Motor group displayed no significant preference, $\chi^2(1, N = 324) = 1$, $p = .317$.

6.3 Inclination to trust in the Maze Game: Choice vs Motor group

To examine the group effect (Choice vs Motor groups) on participants' inclination to trust BeMim and No-BeMim confederates, a binomial logistic mixed-effects model was used, specified as follows: following hint ~ approached confederate * group + (1 | participant ID) + (1 | trial number). The binary outcome "following hint" indicated whether participants adhered to the advice of the confederate they approached for a hint in the Maze Game (coded as 1) or not (coded as 0) across 12 trials. The "approached confederate" variable was coded as BeMim (1) or No-BeMim (0). The results revealed that participants followed the advice from both BeMim and No-BeMim confederate equally often in the Maze Game ($\beta = 0.55$, $p = .104$), with no significant difference between the Motor and Choice groups ($\beta = 0.43$, $p = .296$). Additionally, there was no significant interaction between approaching a BeMim confederate and being in the Motor group ($\beta = -0.62$, $p = .196$; Figure 7B).

6.4 **Inclination to trust in the Maze Game: Choice and Motor groups separately**

To assess participants' inclination to trust BeMim and No-BeMim confederates within the Choice and Motor groups, two separate binomial logistic mixed-effects models were used, one for each group. The models were specified as follows: following hint ~ approached confederate + (1 | participant ID) + (1 | trial number). In the Choice group, participants did not follow the advice of the BeMim confederate significantly more often than that of the No-BeMim confederate (β = 0.56, p = .097). Similarly, in the Motor group, there was no significant difference in following the advice of the BeMim confederate compared to the No-BeMim confederate (β = -0.08, p = .817).

Table S4. *Results for approach tendencies and inclination to trust in the Maze Game.* The table presents the results for binomial logistic mixed-effects models analysing the impact of group (Choice vs Motor) on approach tendencies, and inclination to trust in the Maze Game. Additionally, it details the effects of experimental conditions (BeMim vs No-BeMim) on trust inclination for both Choice and Motor groups.

**Fixed effects**	***Estimate***	***SE***	***z-value***	***p-value***
**Motor vs Choice groups**				
**Approach tendencies in the Maze Game**				
Intercept	0.06	0.16	0.36	.721
Group: Motor	0.06	0.23	0.27	.790
**Inclination to trust in the Maze Game**				
Intercept***	1.60	0.31	5.15	<.001
Group: Motor	0.43	0.41	1.05	.296
Approached confederate: BeMim	0.55	0.34	1.63	.104
Approached confederate and group: BeMim vs Motor	-0.62	0.48	-1.29	.196
**Choice group: Inclination to trust in the Maze Game**				
Intercept***	1.59	0.32	4.94	<.001
Approached confederate: BeMim	0.56	0.34	1.66	.097
**Motor group: Inclination to trust in the Maze Game**				
Intercept***	2.08	0.37	5.69	<.001
Approached confederate: BeMim	-0.08	0.34	-0.23	.817

7. **Additional fNIRS analysis**

In an additional fNIRS analysis, we compared the BeMim task (MC + NC) to the baseline task (MB + NB) and conducted further simple effects tests in regions that showed a significant main effect. It should be noted that the reported p-values are uncorrected for multiple comparisons, and thus these results should be interpreted with caution.

In the Motor group, a significant difference emerged in the left IPS, where hemodynamic responses were lower during the BeMim task (MC + NC) than during baseline (MB + NB) trials, $t(24) = -2.36$, $p = .025$, Cohen's $d = .43$. A follow-up simple effects analysis confirmed reduced hemodynamic activity during MC relative to MB in the left IPS, $t(29) = -2.69$, $p = .012$, Cohen's $d = .49$. As the left IPL is known to underpin goal-oriented hand movements (Buxbaum et al., 2007; Fogassi et al., 2005; Miguel et al., 2021), this result suggests that the region was more engaged when the participant performed a hand-pointing action than when observing the confederate's action, regardless of whether it was mimicry or no-mimicry.

In the Choice group, several significant differences emerged for the BeMim task, with higher haemodynamic changes during (MC + NC) compared with (MB + NB) trials in the right IPL, $t(25) = 2.21$, $p = .037$, Cohen's $d = .43$; the left IPL, $t(29) = 2.50$, $p = .019$, Cohen's $d = .46$; the right IPS, $t(27) = 2.23$, $p = .034$, Cohen's $d = .42$; and the right TPJ, $t(24) = 2.23$, $p = .035$, Cohen's $d = .45$. Follow-up analyses of simple effects revealed increased haemodynamic activity during MC versus MB in the right IPL, $t(25) = 2.63$, $p = .014$, Cohen's $d = .52$; the left IPL, $t(29) = 2.87$, $p = .008$, Cohen's $d = .53$; the right IPS, $t(27) = 2.84$, $p = .008$, Cohen's $d = .54$; and the right TPJ, $t(24) = 2.69$, $p = .013$, Cohen's $d = .25$. Overall, the increased activation in bilateral IPL and the right IPS during the BeMim task (relative to baseline) in the Choice group was mainly driven by higher activity in mimicry-congruent trials, with a slight decrease noted during no-mimicry congruent trials. This pattern aligns with the main findings reported in the manuscript, indicating that the bilateral IPL may track the intention behind the mimicker's choices, consistent with evidence linking the IPL to recognising the goals of observed actions (Grafton & Hamilton, 2007; Patri et al., 2020). Similarly, the right IPS has been associated with tracking others' choices in social decision-making (Suzuki et al., 2015). Although the right TPJ effect was also driven by mimicry-congruent trials relative to baseline, no-mimicry congruent trials increased its activity as well, potentially reflecting broader monitoring of others' choices in line with previous social decision-making (Suzuki et al., 2015) and social influence research (Campbell-Meiklejohn et al., 2010).

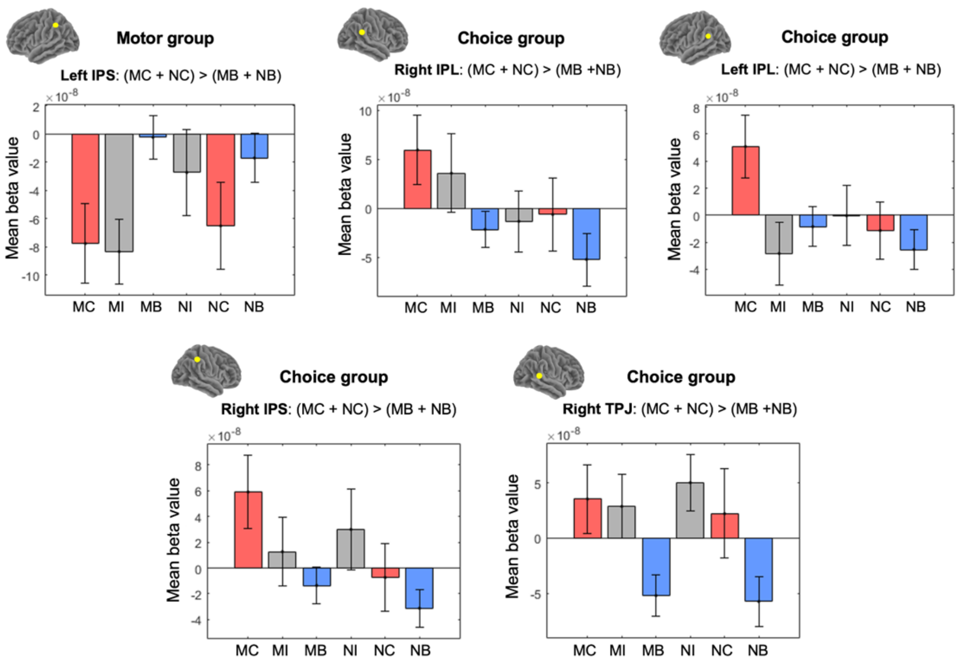

Figure S2. *Additional fNIRS results for Choice and Motor mimicry groups.* MC = BeMim-Congruent trials, NC = No-BeMim-Congruent trials, NI = No-BeMim-Incongruent trials, MI = BeMim-Incongruent trials.

8. Relationship between fNIRS activation and ratings of affiliation

In our first analysis of this dataset, we used a different approach to construct our GLM. Specifically, the timing of the regressors was set up so that each GLM regressor for MC and NC trials spanned the entire duration of the trial, encompassing both the participant's actions and the confederate's responses. For instance, in an MC trial in the Choice group, the model included the period during which the participant examined and chose a painting, as well as the confederate's subsequent mimicry of this choice. We then calculated the simple effect of BeMim (MC > NC) as described in the main text, but with the key difference that each trial was modelled from start to finish. The resulting beta values were used in linear regressions that related the fNIRS BeMim effect to participants' ratings of the confederate's warmth (e.g., "Do you think [confederate] is a warm person?"). By capturing the complete trial rather than focusing on a specific time window, we aimed to reflect the broader, global effects of mimicry. This analysis was moved to the supplementary information following reviewer feedback and is presented here for completeness.

Specifically, we extracted beta values for the BeMim contrast (MC > NC) from MATLAB for each of the 12 ROIs and imported them into R. To create behavioural contrasts, the differences in mean warmth ratings between the BeMim and No-BeMim condition were calculated for each participant. Both the ROI beta values, and the warmth contrast scores were standardised as z-scores within each group to ensure comparability. These standardised contrast scores were then used in linear regression models, conducted separately for the Choice and Motor groups for warmth. Each model

focused on a specific ROI to examine the relationship between brain activity, as measured by fNIRS, and social perception of warmth. The results were corrected for multiple comparisons over 12 ROIs using the FDR method.

In the Choice group, a trend towards significance was observed for perceived warmth in the left STS (β = 0.60, t(21) = 3.14, p = .059), suggesting that increased activity in the left STS was associated with higher perceived warmth. In the Motor group, a significant warmth effect was found in the left IPL (β = 0.56, t(26) = 3.25, p = .038), indicating that increased brain activity in the left IPL was linked to higher perceived warmth.

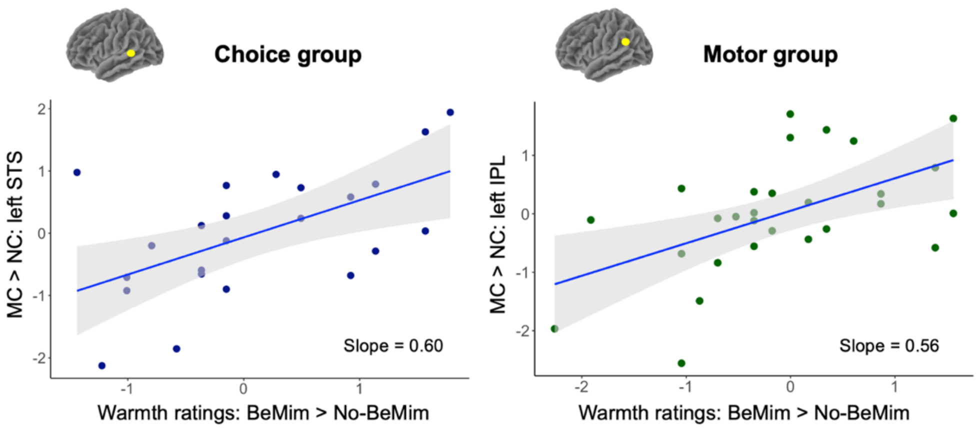

Figure S3. *Relationships between left STS activation and perceived warmth of confederates in the Choice Mimicry group and left IPL activation and perceived warmth in the Motor Mimicry group.*

To further explore these findings across groups, two linear regression models were conducted for the left STS and left IPL, examining the effects of group (Choice vs Motor) and perceived warmth. Both ROI contrasts and the contrast scores for warmth were standardised using z-scores across the entire dataset. The models were then fitted using the formula: ROI value ~ group * perceived warmth, incorporating main effects, as well as two-way interactions. For the left STS, a significant main effect of warmth was observed, (β = 0.70, t(42) = 2.82, uncorrected p = .007), suggesting that higher perceived warmth was associated with increased activation in the left STS. There was no statistically significant difference in the effect of the Choice group compared to the Motor group (β = 0.41, t(42) = 1.37, p = .177). However, a significant interaction between group and warmth was found (β = -0.90, t(42) = -2.87, p = .006), indicating that the relationship between perceived warmth and left STS activation was weaker in the Motor group compared to the Choice group. Similarly, in the left IPL, a significant effect of perceived warmth was identified (β = 0.52, t(54) = 2.89, p = .006), indicating that higher perceived warmth corresponded to increased activation in the left IPL. The Choice group did not show a significant difference from the Motor group (β = 0.52, t(54) = 0.48, p = .635). Furthermore, a significant interaction between group and warmth (β = -0.63, t(54) = -2.30, p = .026)

revealed that the relationship between perceived warmth and left IPL activation was weaker in the Choice group compared to the Motor group.

In the Motor group, the increased activity in the left IPL was associated with higher perceptions of warmth. The left IPL is known to play a role in forming first impressions based on nonverbal cues (Kuzmanovic et al., 2012) and when evaluating co-present observers as supportive rather than unsupportive (Liu et al., 2012). The bilateral IPL is also recognised for its involvement in understanding actions and the their underlying intentions (Caspers et al., 2010; Fogassi et al., 2005). For example, Lamm et al. (2007) demonstrated that the left IPL is activated when participants adopt the perspective of a patient experiencing pain, with activity in the left IPL being correlated with emotional contagion, while activity in the right IPL was observed when participants imagined the patients' feelings. In our study, the increased activity in the left IPL linked to higher perceived warmth in the Motor Group may reflect a self-referential processing mechanism. Specifically, participants may internalise and relate the experience of being mimicked to their own emotional states, fostering a stronger sense of social warmth and connection toward the mimicker.

In the Choice group, we observed that increased activity in the left STS during interactions with confederates correlated with higher ratings of warmth. This aligns with our initial hypothesis that effects of Choice mimicry might be more apparent in social brain networks (STS and TPJ) than in mirror neuron systems. The STS is recognised as a central node in the social brain network, involved in reflecting on others' behaviour and attributing mental states to them, such as opinions (Dasgupta et al., 2017; Gallagher & Frith, 2003). The left STS has also been shown to be involved in person perception of socially relevant information (Zilbovicius et al., 2013) and when interpreting emotional cues from facial or body signals (Cao et al., 2018). Notably, Lee & Harris (2014) found that activity in the left STS is related to perceived warmth when compared to perceived competence or perceptions of computer warmth.

9. Detailed results for fNIRS contrasts

Table S5. *fNIRS results of contrast-driven analyses across 12 ROIs*. **A.** fNIRS results for within-group effects in the Motor group assessed using paired t-tests. **B.** fNIRS results for within-group effects in the Choice group assessed using paired t-tests. **C.** fNIRS results for between-group effects assessed using independent t-tests.

ROI	***t-value***	***df***	***Uncorrected p-value***
**A. fNIRS results for the Motor group**			
**MC > NC**			
Right STS	-0.01	24	.993
Right TPJ	-0.40	20	.691
Right AG	1.17	25	.254

Right IPL	-0.73	25	.470
Right SMG	0.31	25	.756
Right IPS	0.69	27	.497
Left STS	1.56	22	.132
Left TPJ	1.37	26	.183
Left AG	-0.28	24	.781
Left IPL	-0.68	27	.504
Left SMG	0.81	20	.426
Left IPS	-0.34	29	.738
**(MC + MI) > (NC + NI)**			
Right STS	-0.13	24	.899
Right TPJ	-0.24	20	.809
Right AG	-1.26	25	.220
Right IPL	0.29	25	.776
Right SMG	0.41	25	.683
Right IPS	0.74	27	.464
Left STS	0.08	22	.938
Left TPJ	-0.13	26	.900
Left AG	-0.99	24	.331
Left IPL	-1.41	27	.171
Left SMG	-0.43	20	.669
Left IPS	-1.07	29	.295
**(MC + NI) > (NC + MI)**			
Right STS	0.18	24	.859
Right TPJ	-0.37	20	.712
Right AG	0.16	25	.876
Right IPL	-1.45	25	.159
Right SMG	0.03	25	.974
Right IPS	-0.11	27	.911
**Left STS**	**2.19**	**22**	**.039**
**Left TPJ**	**2.31**	**26**	**.029**
Left AG	0.77	24	.450
Left IPL	0.53	27	.602
Left SMG	2.08	20	.051
Left IPS	1.35	29	.188
**(MC + NC) > (MI + NI)**			
**Right STS**	**-2.91**	**24**	**.008**
Right TPJ	-0.75	20	.464
Right AG	-0.91	25	.370
Right IPL	0.35	25	.731
Right SMG	-1.58	25	.126
Right IPS	-1.04	27	.308
Left STS	-1.51	22	.144
Left TPJ	-1.18	26	.250
Left AG	-0.57	24	.572
Left IPL	-0.91	27	.371

Left SMG	-0.05	20	.958
Left IPS	-1.04	29	.306

ROI	*t-value*	*df*	*Uncorrected p-value*
**B. fNIRS results for the Choice group**			
**MC > NC**			
Right STS	0.64	23	.531
Right TPJ	0.32	24	.752
Right AG	1.36	28	.184
Right IPL	1.80	25	.084
Right SMG	0.11	22	.912
Right IPS	1.85	27	.076
Left STS	0.83	22	.414
Left TPJ	0.14	21	.888
Left AG	0.74	24	.464
**Left IPL**	**2.25**	**29**	**.033**
Left SMG	1.47	24	.155
Left IPS	1.57	29	.127
**(MC + MI) > (NC + NI)**			
Right STS	0.18	23	.860
Right TPJ	-0.19	24	.850
Right AG	0.42	28	.679
**Right IPL**	**2.47**	**25**	**.020**
Right SMG	-0.56	22	.581
Right IPS	0.92	27	.365
Left STS	0.05	22	.958
Left TPJ	0.269	21	.790
Left AG	-0.45	24	.658
Left IPL	0.76	29	.451
Left SMG	0.68	24	.501
Left IPS	0.98	29	.336
**(MC + NI) > (NC + MI)**			
Right STS	1.06	23	.302
Right TPJ	0.60	24	.553
Right AG	1.89	28	.070
Right IPL	0.33	25	.743
Right SMG	0.77	22	.452
Right IPS	1.87	27	.073
Left STS	0.94	22	.358
Left TPJ	-0.07	21	.947
Left AG	1.60	24	.123
**Left IPL**	**2.62**	**29**	**.014**
Left SMG	1.29	24	.208

Left IPS	1.39	29	.175
**(MC + NC) > (MI + NI)**			
Right STS	0.51	23	.618
Right TPJ	-0.52	24	.607
Right AG	-0.70	28	.487
Right IPL	0.87	25	.391
Right SMG	-0.68	22	.506
Right IPS	0.22	27	.824
Left STS	0.42	22	.681
Left TPJ	0.09	21	.927
Left AG	0.07	24	.941
Left IPL	1.89	29	.069
Left SMG	1.65	24	.111
Left IPS	0.85	29	.403

ROI	***t-value***	***df***	***Uncorrected p-value***
**C. fNIRS results for Motor vs Choice**			
**MC > NC**			
Right STS	-0.59	47	.561
Right TPJ	-0.45	44	.653
Right AG	-0.20	53	.841
Right IPL	-1.89	50	.065
Right SMG	0.11	47	.909
Right IPS	-1.10	54	.278
Left STS	0.64	44	.528
Left TPJ	0.68	47	.500
Left AG	-0.77	48	.446
**Left IPL**	**-2.05**	**56**	**.045**
Left SMG	-0.64	44	.524
Left IPS	-1.46	58	.149
**(MC + MI) > (NC + NI)**			
Right STS	-0.22	47	.826
Right TPJ	0.03	44	.980
Right AG	0.68	53	.498
Right IPL	-1.57	50	.123
Right SMG	0.70	47	.485
Right IPS	-0.13	54	.893
Left STS	0.04	44	.968
Left TPJ	-0.29	47	.776
Left AG	-0.30	48	.768
Left IPL	-1.50	56	.139
Left SMG	-0.08	44	.440
Left IPS	-1.45	58	.153

**(MC + NI) > (NC + MI)**			
Right STS	-0.79	47	.433
Right TPJ	-0.67	44	.505
Right AG	-1.32	53	.192
Right IPL	-1.11	50	.273
Right SMG	-0.69	47	.495
Right IPS	-1.62	54	.112
Left STS	0.79	44	.431
Left TPJ	1.37	47	.178
Left AG	-0.90	48	.371
Left IPL	-1.52	56	.133
Left SMG	-0.07	44	.947
Left IPS	-0.68	58	.502
**(MC + NC) > (MI + NI)**			
**Right STS**	**-2.11**	**47**	**.040**
Right TPJ	-0.08	44	.938
Right AG	-0.08	53	.935
Right IPL	-0.40	50	.688
Right SMG	-0.41	47	.682
Right IPS	-0.87	54	.389
Left STS	-1.34	44	.186
Left TPJ	-0.88	47	.383
Left AG	-0.43	48	.671
Left IPL	-1.98	56	.053
Left SMG	-1.01	44	.318
Left IPS	-1.27	58	.208

10. Multiple comparisons, sample power and required sample size

To address uncorrected findings due to sample size, we applied FDR correction across all 12 ROIs separately for the Choice and Motor groups, leading to previously significant results becoming insignificant. We then estimated the power of these corrected results and calculated the required sample sizes to achieve significance ($p < .05$) with a target power of 0.8, accounting for multiple comparisons. By including power calculations, we aim for transparency regarding the limitations of our current sample size and offer guidance for future research in this domain.

8.1 BeMim effect: MC > NC

Within the Choice group, the simple BeMim effect (MC > NC) initially showed a significant difference in the left IPL ($p = .033$). After applying FDR correction across 12 ROIs, this finding became non-significant ($p = .338$). With the current sample size of 30 participants, the corrected result has a power of 0.35. Reaching the desired power of 0.8 would require a sample size of 95 participants.

8.2 BeMim condition effect: (MC + MI) > (NC + NI)

For the main BeMim condition effect in the Choice group, we initially identified a significant difference in the right IPL (p = .020). Following FDR correction, this result was no longer significant (p = .246). With a sample size of 26 participants, the corrected effect has a power of 0.40. Achieving a power of 0.8 would necessitate 68 participants.

8.3 Interaction effect: (MC + NI) > (NC + MI)

For the Motor group, a significant difference was first noted in the left STS (p = .039), which became non-significant after FDR correction (p = .202). With 23 participants, the corrected result has a power of 0.33. A sample size of 77 participants would be required to reach a power of 0.8.

A further significant finding in the Motor group emerged in the left TPJ (p = .029), which similarly lost significance after FDR correction (p = .202). Here, the current sample size of 27 participants yields a power of 0.36. Increasing the sample size to 81 participants would be necessary for a power of 0.8.

In the Choice group, the left IPL was originally significant (p = .014), then became non-significant (p = .166) post-FDR correction. With a sample size of 30 participants, the corrected result has a power of 0.45, indicating that 70 participants would be needed to achieve a power of 0.8.

8.4 Congruency effect: (MC + NC) > (MI + NI)

For the main congruency effect in the Motor group, a significant difference was initially observed in the right STS (p = .008), which became non-significant after FDR correction (p = .092). With 25 participants, the corrected result has a power of 0.52. Increasing the sample to 48 participants would be required to reach the target power of 0.8.

References

Aron, A., Aron, E. N., & Smollan, D. (1992). Inclusion of Other in the Self Scale and the structure of interpersonal closeness. *Journal of Personality and Social Psychology*, *63*(4), 596–612. https://doi.org/10.1037/0022-3514.63.4.596

Bagby, R. M., Parker, J. D. A., & Taylor, G. J. (1994). The twenty-item Toronto Alexithymia scale—I. Item selection and cross-validation of the factor structure. *Journal of Psychosomatic Research*, *38*(1), 23–32. https://doi.org/10.1016/0022-3999(94)90005-1

Brass, M., Ruby, P., & Spengler, S. (2009). Inhibition of imitative behaviour and social cognition. *Philosophical Transactions of the Royal Society B: Biological Sciences*, *364*(1528), 2359–2367. https://doi.org/10.1098/rstb.2009.0066

Brown, E. J., Turovsky, J., Heimberg, R. G., & Juster, H. R. (n.d.). *Validation of the Social Interaction Anxiety Scale and the Social Phobia Scale Across the Anxiety Disorders*.

Brown, E., Turovsky, J., Heimberg, R., Juster, H., Brown, T., & David, B. (1997). Validation of the Social Interaction Anxiety Scale and the Social Phobia Scale Across the Anxiety Disorders. *Psychological Assessment*, *9*, 21–27. https://doi.org/10.1037/1040-3590.9.1.21

Buxbaum, L. J., Kyle, K., Grossman, M., & Coslett, B. (2007). Left Inferior Parietal Representations for Skilled Hand-Object Interactions: Evidence from Stroke and Corticobasal Degeneration. *Cortex*, *43*(3), 411–423. https://doi.org/10.1016/S0010-9452(08)70466-0

Campbell-Meiklejohn, D. K., Bach, D. R., Roepstorff, A., Dolan, R. J., & Frith, C. D. (2010). How the Opinion of Others Affects Our Valuation of Objects. *Current Biology*, *20*(13), 1165–1170. https://doi.org/10.1016/j.cub.2010.04.055

Cao, L., Xu, J., Yang, X., Li, X., & Liu, B. (2018). Abstract Representations of Emotions Perceived From the Face, Body, and Whole-Person Expressions in the Left Postcentral Gyrus. *Frontiers in Human Neuroscience*, *12*. https://doi.org/10.3389/fnhum.2018.00419

Caspers, S., Zilles, K., Laird, A. R., & Eickhoff, S. B. (2010). ALE meta-analysis of action observation and imitation in the human brain. *NeuroImage*, *50*(3), 1148–1167. https://doi.org/10.1016/j.neuroimage.2009.12.112

Chambon, V., Domenech, P., Jacquet, P. O., Barbalat, G., Bouton, S., Pacherie, E., Koechlin, E., & Farrer, C. (2017). Neural coding of prior expectations in hierarchical intention inference. *Scientific Reports*, *7*(1), 1278. https://doi.org/10.1038/s41598-017-01414-y

Cloutier, J., & Gyurovski, I. (2013). Intraparietal sulcus activity during |g explicit self-referential social status judgments about others. *International Journal of Psychological Research*, *6*, 68–79. https://doi.org/10.21500/20112084.721

Dasgupta, S., Tyler, S. C., Wicks, J., Srinivasan, R., & Grossman, E. D. (2017). Network Connectivity of the Right STS in Three Social Perception Localizers. *Journal of Cognitive Neuroscience*, *29*(2), 221–234. https://doi.org/10.1162/jocn_a_01054

Davis, M. H. (2011). *Interpersonal Reactivity Index* [Dataset]. https://doi.org/10.1037/t01093-000

De Felice, S., Hakim, U., Gunasekara, N., Pinti, P., Tachtsidis, I., & Hamilton, A. (2024). *Having a chat and then watching a movie: How social interaction synchronises our brains during co-watching*. https://doi.org/10.1093/oons/kvae006

Farmer, H., Hertz, U., & Hamilton, A. F. de C. (2019). The neural basis of shared preference learning. *Social Cognitive and Affective Neuroscience*, *14*(10), 1061–1072. https://doi.org/10.1093/scan/nsz076

Farrer, C., Frey, S. H., Van Horn, J. D., Tunik, E., Turk, D., Inati, S., & Grafton, S. T. (2008). The Angular Gyrus Computes Action Awareness Representations. *Cerebral Cortex*, *18*(2), 254–261. https://doi.org/10.1093/cercor/bhm050

Fogassi, L., Ferrari, P. F., Gesierich, B., Rozzi, S., Chersi, F., & Rizzolatti, G. (2005). Parietal Lobe: From Action Organization to Intention Understanding. *Science*, *308*(5722), 662–667. https://doi.org/10.1126/science.1106138

Gallagher, H. L., & Frith, C. D. (2003). Functional imaging of 'theory of mind'. *Trends in Cognitive Sciences*, *7*(2), 77–83. https://doi.org/10.1016/S1364-6613(02)00025-6

Grafton, S. T., & de C. Hamilton, A. F. (2007). Evidence for a distributed hierarchy of action representation in the brain. *Human Movement Science*, *26*(4), 590–616. https://doi.org/10.1016/j.humov.2007.05.009

Gratch, J., Wang, N., Gerten, J., Fast, E., & Duffy, R. (2007). Creating Rapport with Virtual Agents. In C. Pelachaud, J.-C. Martin, E. André, G. Chollet, K. Karpouzis, & D. Pelé (Eds.), *Intelligent Virtual Agents* (pp. 125–138). Springer. https://doi.org/10.1007/978-3-540-74997-4_12

Hale, J., Payne, M. E., Taylor, K. M., Paoletti, D., & De C Hamilton, A. F. (2018). The virtual maze: A behavioural tool for measuring trust. *Quarterly Journal of Experimental Psychology*, *71*(4), 989–1008. https://doi.org/10.1080/17470218.2017.1307865

Harker, L., & Keltner, D. (2001). Expressions of positive emotion in women's college yearbook pictures and their relationship to personality and life outcomes across adulthood. *Journal of Personality and Social Psychology*, *80*(1), 112–124. https://doi.org/10.1037/0022-3514.80.1.112

Kuzmanovic, B., Bente, G., von Cramon, D. Y., Schilbach, L., Tittgemeyer, M., & Vogeley, K. (2012). Imaging first impressions: Distinct neural processing of verbal and nonverbal social information. *NeuroImage*, *60*(1), 179–188. https://doi.org/10.1016/j.neuroimage.2011.12.046

Lamm, C., Batson, C. D., & Decety, J. (2007). The Neural Substrate of Human Empathy: Effects of Perspective-taking and Cognitive Appraisal. *Journal of Cognitive Neuroscience*, *19*(1), 42–58. https://doi.org/10.1162/jocn.2007.19.1.42

Lee, V. K., & Harris, L. T. (2014). Sticking with the nice guy: Trait warmth information impairs learning and modulates person perception brain network activity. *Cognitive, Affective, & Behavioral Neuroscience*, *14*(4), 1420–1437. https://doi.org/10.3758/s13415-014-0284-9

Liu, T., Saito, H., Oi, M., & Pelowski, M. (2012). Appraisal of a copresent observer as supportive activates the left inferior parietal lobule: A near-infrared spectroscopy study using a driving video game. *NeuroReport*, *23*(14), 835. https://doi.org/10.1097/WNR.0b013e328357bb3b

Mainieri, A. G., Heim, S., Straube, B., Binkofski, F., & Kircher, T. (2013). Differential role of the Mentalizing and the Mirror Neuron system in the imitation of communicative gestures. *NeuroImage*, *81*, 294–305. https://doi.org/10.1016/j.neuroimage.2013.05.021

Mengotti, P., Corradi-Dell'Acqua, C., & Rumiati, R. I. (2012). Imitation components in the human brain: An fMRI study. *NeuroImage*, *59*(2), 1622–1630. https://doi.org/10.1016/j.neuroimage.2011.09.004

Miguel, H. O., Condy, E. E., Nguyen, T., Zeytinoglu, S., Blick, E., Bress, K., Khaksari, K., Dashtestani, H., Millerhagen, J., Shahmohammadi, S., Fox, N. A., & Gandjbakhche, A. (2021). Cerebral hemodynamic response during a live action-observation and action-execution task: A fNIRS study. *PLoS ONE*, *16*(8), e0253788. https://doi.org/10.1371/journal.pone.0253788

Patri, J.-F., Cavallo, A., Pullar, K., Soriano, M., Valente, M., Koul, A., Avenanti, A., Panzeri, S., & Becchio, C. (2020). Transient Disruption of the Inferior Parietal Lobule Impairs the Ability to Attribute Intention to Action. *Current Biology*, *30*(23), 4594-4605.e7. https://doi.org/10.1016/j.cub.2020.08.104

Suzuki, S., Adachi, R., Dunne, S., Bossaerts, P., & O'Doherty, J. P. (2015). Neural Mechanisms Underlying Human Consensus Decision-Making. *Neuron*, *86*(2), 591–602. https://doi.org/10.1016/j.neuron.2015.03.019

Zilbovicius, M., Saitovitch, A., Popa, T., Rechtman, E., Diamandis, L., Chabane, N., Brunelle, F., Samson, Y., & Boddaert, N. (2013). *Autism, social cognition and superior temporal sulcus*. *2013*. https://doi.org/10.4236/ojpsych.2013.32A008
